# Whole-Genome Cardiac DNA Methylation Fingerprint and Gene Expression Analysis Provide New Insights in the Pathogenesis of Chronic Chagas Disease Cardiomyopathy

**DOI:** 10.1093/cid/cix506

**Published:** 2017-05-30

**Authors:** Laurie Laugier, Amanda Farage Frade, Frederico Moraes Ferreira, Monique Andrade Baron, Priscila Camillo Teixeira, Sandrine Cabantous, Ludmila Rodrigues Pinto Ferreira, Laurence Louis, Vagner Oliveira Carvalho Rigaud, Fabio Antônio Gaiotto, Fernando Bacal, Pablo Pomerantzeff, Edimar Bocchi, Jorge Kalil, Ronaldo Honorato Barros Santos, Edecio Cunha-Neto, Christophe Chevillard

**Affiliations:** 1 Aix Marseille Université, Génétique et Immunologie des Maladies Parasitaires, Unité Mixte de Recherche S906, INSERM U906, Marseille, France;; 2 Laboratory of Immunology, Heart Institute, University of São Paulo School of Medicine,; 3 Institute for Investigation in Immunology (iii), INCT,; 4 Department of Bioengineering, Brazil University, and; 5 Health Sciences, University of Santo Amaro, São Paulo, Brazil;; 6 Aix Marseille Université, Génétique médicale et génomique fonctionnelle (Plateforme Génomique et Transcriptomique), Unité Mixte de Recherche S910, INSERM U910, Marseille, France; Divisions of; 7 Pathology and; 8 Heart Transplantation, and; 9 Heart Failure Unit, Heart Institute, University of São Paulo School of Medicine, and; 10 Division of Clinical Immunology and Allergy, University of São Paulo School of Medicine, Brazil

**Keywords:** epigenetic, methylation, cardiomyopathy, Chagas, gene expression

## Abstract

**Background:**

Chagas disease, caused by the protozoan *Trypanosoma cruzi*, is endemic in Latin America and affects 10 million people worldwide. Approximately 12000 deaths attributable to Chagas disease occur annually due to chronic Chagas disease cardiomyopathy (CCC), an inflammatory cardiomyopathy presenting with heart failure and arrythmia; 30% of infected subjects develop CCC years after infection. Genetic mechanisms play a role in differential progression to CCC, but little is known about the role of epigenetic modifications in pathological gene expression patterns in CCC patients’ myocardium. DNA methylation is the most common modification in the mammalian genome.

**Methods:**

We investigated the impact of genome-wide cardiac DNA methylation on global gene expression in myocardial samples from end-stage CCC patients, compared to control samples from organ donors.

**Results:**

In total, 4720 genes were differentially methylated between CCC patients and controls, of which 399 were also differentially expressed. Several of them were related to heart function or to the immune response and had methylation sites in their promoter region. Reporter gene and in silico transcription factor binding analyses indicated promoter methylation modified expression of key genes. Among those, we found potassium channel genes *KCNA4* and *KCNIP4*, involved in electrical conduction and arrythmia, *SMOC2*, involved in matrix remodeling, as well as enkephalin and *RUNX3*, potentially involved in the increased T-helper 1 cytokine-mediated inflammatory damage in heart.

**Conclusions:**

Results support that DNA methylation plays a role in the regulation of expression of pathogenically relevant genes in CCC myocardium, and identify novel potential disease pathways and therapeutic targets in CCC.

## Summary

DNA methylation analysis on global gene expression in myocardial samples from patients with end-stage chronic Chagas cardiomyopathy leads to the identification of novel potential disease pathways and therapeutic targets linked to electrical conduction or immune response modulation.

Chagas disease is a neglected tropical disease caused by the protozoan *Trypanosoma cruzi* and transmitted mainly by hematophagous insect vectors in endemic areas of South and Central America [1]. It is now a concern in nonendemic industrialized countries due to immigration, and causes economic losses in excess of 7 billion dollars. With time, approximately 30% of infected patients develop chronic Chagas disease cardiomyopathy (CCC), whereas 60%–70% of infected individuals remain asymptomatic for life. CCC frequently leads to severe inflammatory dilated cardiomyopathy, where heart failure and arrythmia are the main causes of death. The mechanisms underlying CCC development and progression are still not fully understood. The intensity of myocardial inflammation, fueled by chemokine-driven interferon gamma (IFN-γ)–producing helper T-cell type 1 (Th1) *T. cruzi*–reactive and heart-autoreactive T cells and macrophages, correlates with disease severity. Along with myocarditis, the ensuing heart fiber damage, myocardial hypertrophy, and fibrosis contribute to CCC pathogenesis and progression [2–5]. The prognosis of CCC is worse than that of other dilated cardiomyopathies, and genetic factors have been associated with susceptibility to Chagas disease and CCC [6–9]. Likewise, our group has shown profound changes in the gene expression profile of CCC myocardium [10]. Epigenetic changes modulate gene expression without alterations in DNA sequence, respond to environmental and context-specific stimuli, and play a key role in many physiological phenomena and disease states. Our group has observed epigenetic dysregulation of noncoding RNAs in hearts of CCC patients and *T. cruzi*–infected mice [11–14], where microRNAs regulate expression of pathogenetically relevant target genes [14]. DNA methylation is the most common epigenetic modification in the mammalian genome. DNA methylation mainly inhibits gene expression, but can also be positively correlated with expression. Altered DNA methylation patterns in the myocardium of patients with idiopathic dilated cardiomyopathy cause altered expression of genes involved in heart failure [15–17].

In the present study, we investigated for the first time the impact of cardiac DNA methylation on gene expression in patients suffering from CCC. To do so, we identified genes characterized by both an altered methylation status and differential expression levels. We then studied whether the methylation variations in promoter subregions could explain variation of expression using gene reporter assays.

## METHODS

### Ethical Considerations

The protocol was approved by the institutional review boards of the University of São Paulo School of Medicine and INSERM (French National Institute of Health and Medical Research). Written informed consent was obtained from the patients or family members. All experimental methods comply with the Helsinki Declaration.

### Patients and Tissue Collection

Human left ventricular free wall heart tissue samples were obtained from patients with end-stage heart failure CCC at the time of heart transplantation (n = 25). CCC patients underwent serological diagnosis of *T. cruzi* infection and standard electrocardiography and echocardiography, and tissues were subject to histopathological assessment [13]. Biopsies from controls (n = 7) were obtained from healthy hearts of organ donors having no suitable recipient (Table 1).

**Table 1.** Characteristics of the Human Left Ventricular Free Wall Heart Tissue Samples Used in This Study

Project Number	Form	EF (%)	Age	Sex	Transcriptome Analysis	Illumina Methylation BeadChip	Pyrosequencing
EBS	CCC	12	32	M	x	x	x
NSR	CCC	15	49	F	x	x	x
MGS	CCC	20	61	F			x
BHAN	CCC	20	15	M			x
SCS	CCC	17	59	M	x	x	x
ECA	CCC	19	32	F			x
VTL	CCC	19	41	M			x
APA	CCC	20	60	F	x	x	x
MCRS	CCC	20	45	F	x	x	x
MERS	CCC	20	39	F			x
MSS	CCC	20	46	F			x
GMS	CCC	20	58	M		x	x
ISM	CCC	20	39	M		x	x
OMG	CCC	21	49	M		x	x
MAP	CCC	23	50	F	x	x	x
EPG	CCC	2	41	M			x
JRJ	CCC	23	51	M		x	x
LRJ	CCC	25	66	F			x
HBO	CCC	25	36	M	x	x	x
PMG	CCC	29	57	M	x	x	x
ABG	CCC	30	64	F			x
ZMC	CCC	36	54	F	x	x	x
JAB	CCC	55	41	M			x
AAF2	CCC	64	60	M	x	x	x
JMS	CCC	7	50	M			x
EMBT	Control		25	M	x	x	x
LO	Control		46	M	x	x	x
ESS	Control		22	M	x	x	x
ZFS	Control			M	x	x	x
FJR	Control		28	M	x	x	x
MBFM	Control		17	M	x	x	x
3557	Control			M	x	x	x

First, we performed the gene expression on 10 CCC patients and 7 controls (all the tissue samples available at that time). Then, we performed the methylation analysis using the array. Experiments were done on 14 CCC patients and 7 controls (all the tissue samples available at this time). Finally, the pyrosequencing reactions were done on a larger set of samples (25 CCC patients and 7 controls) as the tissue recruitment was ongoing.

Abbreviations: CCC, chronic Chagas disease cardiomyopathy; EF, ejection fraction; F, female; M, male.

### RNA Extraction From Heart Tissue Biopsies

Heart tissue samples (20–30 mg) were cleared from pericardial fat, crushed with ceramic beads (CK14, diameter 1.4 mm) in 350 µL of RLT lysis buffer supplemented with 3.5 µL of β-mercapto-ethanol. Total RNA was extracted from biopsies using the RNeasy Mini Kit adapted with Trizol. RNA quantity was measured with a 2100 Bioanalyser.

### Whole-Transcriptome Analysis

Whole-genome expression analysis was done on SurePrint G3 Human GeneExpression v1 8x60K arrays (Agilent Technologies, Les Ulis, France) following the manufacturer’s standard protocol. Microarray analyses and signal normalization were done with GeneSpring software (11.5.1). The *P* values were obtained with Student *t* test and adjusted with Benjamini-Hochberg correction. The cutoff levels were adjusted *P* value <.05 and an absolute fold change >2. Gene expression data files are available under the Gene Expression Omnibus accession number GSE84796.

### DNA Extraction From Heart Tissue Biopsies

Heart tissue samples were crushed twice with ATL buffer. After proteinase K treatment, DNA was extracted with QIAamp DNA Mini Kit (Qiagen, Courtaboeuf, France) according to the manufacturer’s recommendations.

### Genome-wide DNA Methylation Profiling

DNAs, extracted from whole tissue, were bisulfite converted and amplified with elongation of primers with EZ DNA methylation kit (ZymoResearch, Saint Marcel, France). Amplified DNAs were fragmented and hybridized in beads (Infinium Human Methylation 450 Bead Chip, Illumina, San Diego, California). The threshold *P* value was 1 × 10^-7^, taking into account the effect of multiple testing.

### Pyromark CpG Assays

Reactions with PyroMark assays, EpiTect Bisulfite procedures, and PyroMark gold Q24 kit sequencing were performed according to manufacturer’s instructions (Qiagen, Cortaboeuf, France).

### Plasmid Vector Synthesis

Synthetized promoter region fragments were cloned in the pCpG‐free-basic-Lucia vector (2944 bp) (Invivogen, Toulouse, France). Constructs were verified by Sanger sequencing (Supplementary Table 1).

### Bacterial Strain Plasmid Amplification and Purification

Plasmids with insert were spread with One Shot PIR1 Chemically Competent *Escherichia coli* in the presence of zeocin (ThermoFisher Scientific, Saint Aubin, France). The Renilla control vector (pRL-TK vector; Promega, Charbonnières Les Bains, France) was spread with *E. coli* PIR1 cells. Plasmids with insert or Renilla plasmid were then purified (plasmid Midi Kit, Qiagen).

### Plasmid Methylation

Plasmid constructs encoding short sequences of promoter regions containing CpG sites in genes of interest were methylated using MssI methylase (New England Biolabs, Evry, France) according to the manufacturer’s recommendation.

### Human Cell Culture

HEK293 cells were grown in Dulbecco’s modified Eagle’s medium (DMEM; Lonza, Levallois Perret, France) supplemented with 10% fetal calf serum (FCS), 2% l-glutamine, 1% Fungizone 100X (all from ThermoFisher Scientific), and 0.5% penicillin-streptomycin (Life Technologies). The AC16 cardiomyocyte cell line was grown in DMEM-F12 with 12.5% FCS and the same supplements. Cells were grown at 37°C in a 5% CO_2_ moisturized atmosphere [18].

### Transfections

HEK293 or AC16 cells were seeded in 24-well plates at an initial density of 5 × 10^4^ cells per well, with 400 μL of supplemented medium. Cells were incubated overnight and transfections were done with transfection agent DreamfectGold for HEK293 cells and PolyMagNeo for AC16 cells (OZBiosciences, Marseille, France). Transfected cells were incubated for 24 hours at 37°C in a 5% CO_2_ moisturized atmosphere.

### Promoter Luciferase Assays

Cell lysates were assayed for Lucia luciferase and Renilla luciferase activity using the Dual-Luciferase Reporter Assay System (Promega) on a Glomax Multi Luminometer (Promega) according to the manufacturer’s instructions.

## RESULTS

### Methylation Profile

**Figure 1.**
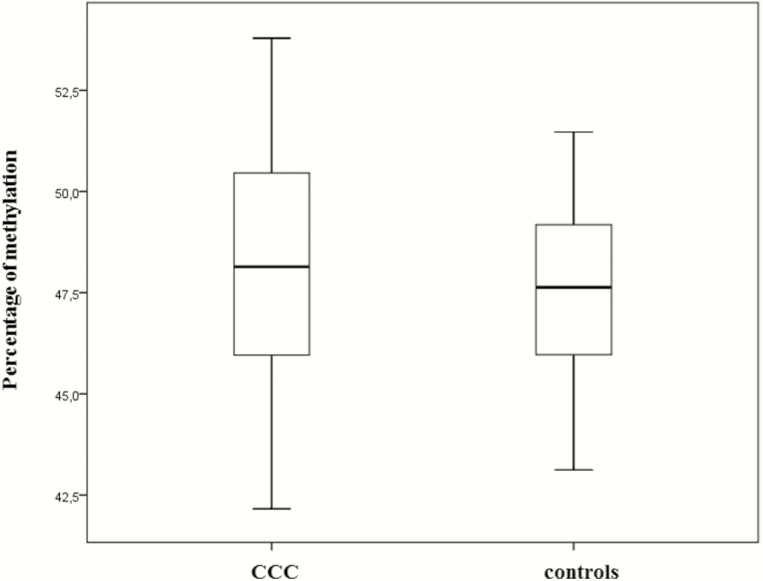
Percentage of methylation in heart biopsy samples. Mean DNA methylation levels in all tested sites in samples from 14 patients with chronic Chagas disease cardiomyopathy (CCC) and 7 controls. Upper and lower hinges of the box, 75th and 25th percentiles, respectively; whiskers represent highest and lowest values.

There was no difference in the mean methylation level between CCC and controls (Figure 1). We identified 7595 differentially methylated sites between CCC and controls with a *P* value <10^-7^. Of these sites, 2649 (35%) were undermethylated and 4946 (65%) were overmethylated in CCC myocardium. For one CpG, the delta β-value between CCC patients and controls is the difference between the methylation mean of the CCC and the methylation mean of the controls intensity of the methylated allele / (intensity of the unmethylated allele + intensity of the methylated allele). The absolute delta β-values ranged from 3.78% to 43.6%.

**Figure 2.**
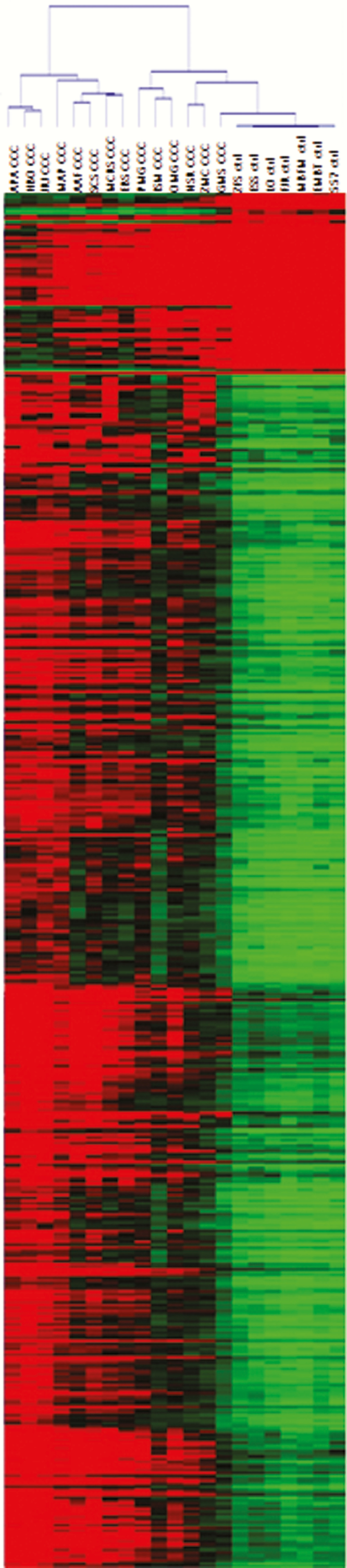
Unsupervised hierarchical clustering of methylation in 14 patients with severe chronic Chagas disease cardiomyopathy (CCC) and 7 controls. Unsupervised hierarchical clustering of DNA methylation based on the top 500 CpG probes. Each row is a CpG site and each column is a sample. The DNA-methylation delta β-values are represented using a color scale from green (low DNA methylation) to red (high DNA methylation).

**Figure 3.**
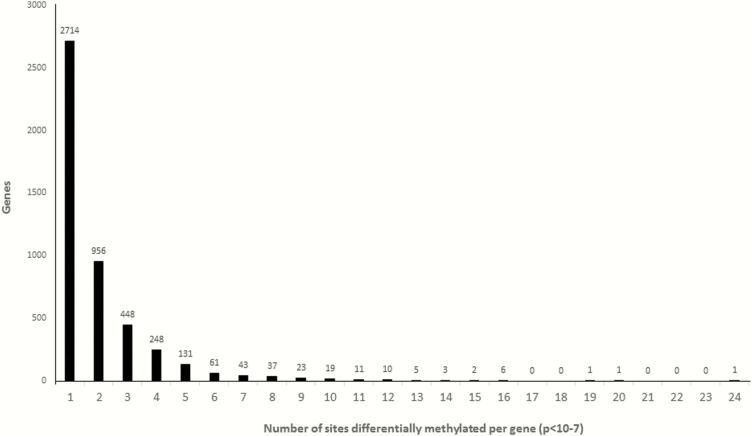
Distribution of CpG sites in differentially methylated genes. A total of 4720 genes were differentially methylated between patients with chronic Chagas disease cardiomyopathy and controls; each has between 1 and 24 CpG sites (*P* < 10^-7^).

Hierarchical clustering analysis based on the most significant CpG probes showed clear discrimination between the groups (Figure 2). The methylation rate of a gene (coding region plus 10 kb upstream and downstream regions) corresponds to the average methylation rate of all differentially methylated sites within it. A total of 7595 differentially methylated CpG sites were located in 4720 genes. Among 4720 differentially methylated genes, 1772 genes (38%) were undermethylated and 2948 genes (62%) were overmethylated in CCC. The numbers differential methylation of CpG sites per gene are shown in Figure 3.

### Genes Differentially Expressed Between Chagas Disease Cardiomyopathy and Controls

Transcriptome analysis was performed on the same myocardial samples (Table 1). A total of 1535 genes were differentially expressed, of which 438 were downregulated (28%) and 1097 upregulated (72%), and its results were in line with previously published data, but the present whole-genome analysis identified many more differentially expressed immune-related genes than the previous cardiovascular gene–based microarray study [10]. To verify the accuracy of the microarray results, 44 genes differentially expressed in CCC biopsies were selected for validation by quantitative reverse-transcription polymerase chain reaction, of which 36 (80%) were confirmed (Supplementary Table 2).

### Comparison of Genome-wide Methylation and Expression Profiles Between Chagas Disease Cardiomyopathy Patients and Controls

**Figure 4.**
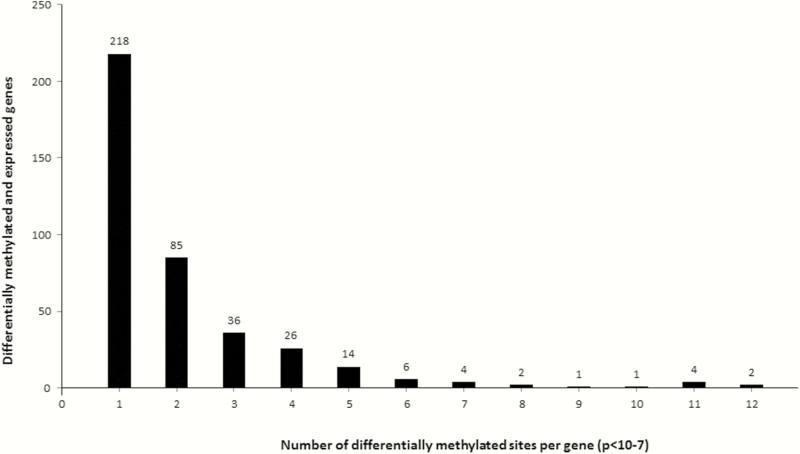
Distribution of CpG sites in differentially expressed genes and differentially methylated genes. Three hundred ninety-nine genes are differentially expressed and methylated in the myocardial samples of 10 patients with chronic Chagas disease cardiomyopathy and 7 controls (1 to 12 CpG sites, *P* < 10^-7^). Thirty-four of these genes are differentially expressed and contain at least 5 differentially methylated sites irrespective of their position (arbitrary cutoff). Among them, 23 play a role in immune response or heart functions. The remaining genes that contain >5 differentially methylated sites and not further investigated are *C16orf54*, *PITX1*, *PRLHR*, *PTPRVP*, *SLFN12L*, *STAG3*, *SYTL1*, *TMC8*, *WDFY4*, *XAF1*, and *ZNRD1-AS1*.

Methylation analysis was done 14 CCC patients and 7 controls whereas expression analysis was done on 10 CCC patients and 7 controls (Table 1). We identified 399 genes that were simultaneously differentially expressed and differentially methylated between CCC and controls (Supplementary Table 3); the number of differentially methylated CpG sites per gene is shown in Figure 4. Most of these genes encode membrane components or receptors, and the main biological processes involved are linked to the immune response (Supplementary Tables 4 and 5). Some of these differentially expressed genes were previously associated to Chagas disease (eg, *IL7*, *CCR7*, *CCL19*, *GATA4*, *HLA-DPB1*). Moreover, some contractile and metabolism genes present the same pattern in a previous study [10]. However, no polymorphism in or around these genes was previously associated to susceptibility to CCC (except for HLA locus).

**Figure 5.**
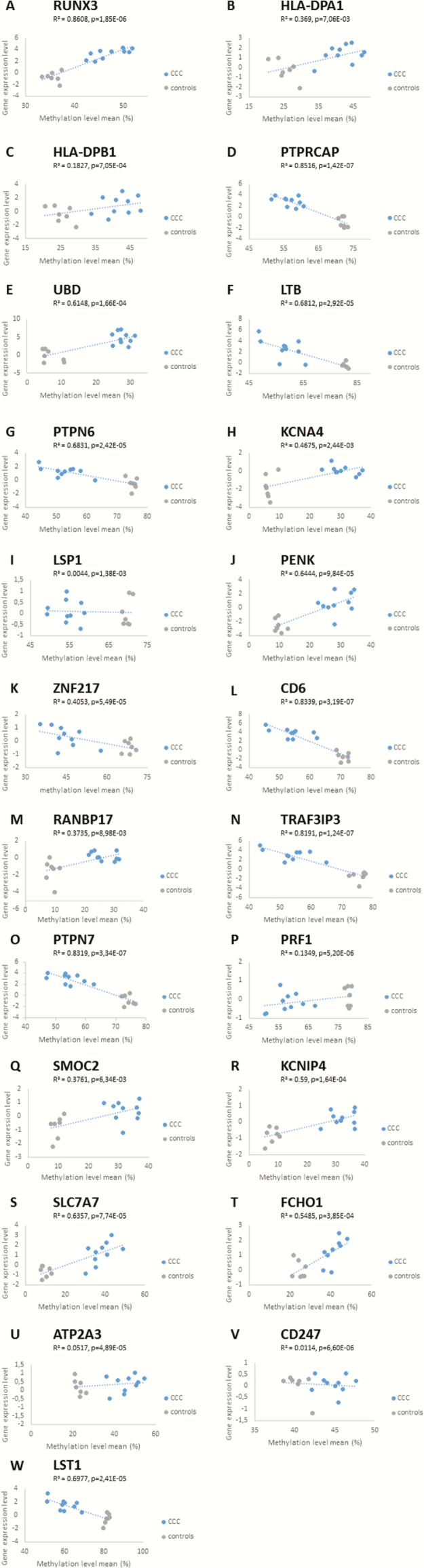
*A–W*, Relationship between gene expression levels and methylation. Correlation between messenger RNA expression and the mean methylation percentage of all CpG sites in 23 selected genes in patients with chronic Chagas disease cardiomyopathy (CCC) and controls, according to linear regression analysis. *P* < .05 was considered statistically significant. DNA methylation in promoters is closely linked to downstream gene repression. DNA methylation may affect the affinity of transcription factors for their binding sites. An average promoter methylation is usually used in studies, whereas recent results suggested that methylation of individual cytosines can also be important. However, it is absolutely not obvious if this dogma may be applied to CpG sites located in the gene body or 3ʹ region. This may explain why we detected direct or inverse correlations between our expression level and methylation level.

We found that 34 genes are both differentially expressed and contain at least 5 differentially methylated sites, irrespective of their position; 23 of the 34 play a role in immune response or heart functions and were further investigated. Correlation between expression and methylation was found to be significant for all the genes (Figure 5). An inverse correlation was observed between the expression level and the percentage of methylation for 10 of these 23 genes. For the 399 genes, linear regression analysis indicated a significant correlation between expression level (eQTL) and methylation level (mQTL) for most genes (386 with *P* < .05). All *P* values are provided in Supplementary Table 6.

### Validation of the Methylation Profiles Using Pyrosequencing

Methylation level confirmation was done on the same DNAs used for the whole methylation analysis (14 CCC patients and 7 controls) plus DNAs extracted from additional left ventricular free wall heart tissue samples from 11 CCC patients (Table 1). The screening focused on the 157 CpG sites located in these 23 genes; 122 Pyromark assays were available (Qiagen). Eighty-six assays (70.5%) passed the quality controls. For statistical analysis the cutoff value was set at 5.10^-4^ (0.05/122 is close to 5.10^-4^). In our tissue sample collection, 68 of 86 (79%) assays led to significant results (Supplementary Table 7).

### Effect of Promoter Methylation on Gene Expression: Functional Study

**Figure 6.**
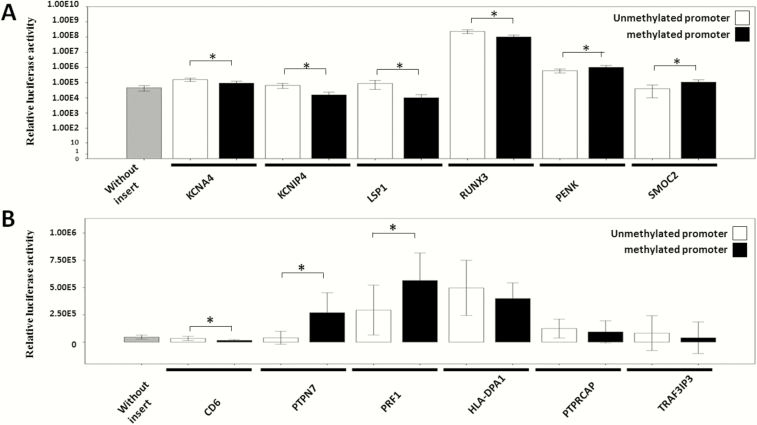
Effect of vector backbone CpG methylation on a CpG-free promoter by reporter gene analysis in transient transfections of the HEK 293 cell line. *A* and *B*, Gray bar represents reporter gene expression in the absence of promoter insert. White bars represent vector with unmethylated insert. Black bars represent vector with methylated insert (±2 standard deviations). Experimental points were done in quadruplicate and experiments were repeated 4 times. For statistical analysis, a cutoff value of .004 was used. *The difference is statistically significant (the *P* value is under the cutoff value).

**Figure 7.**
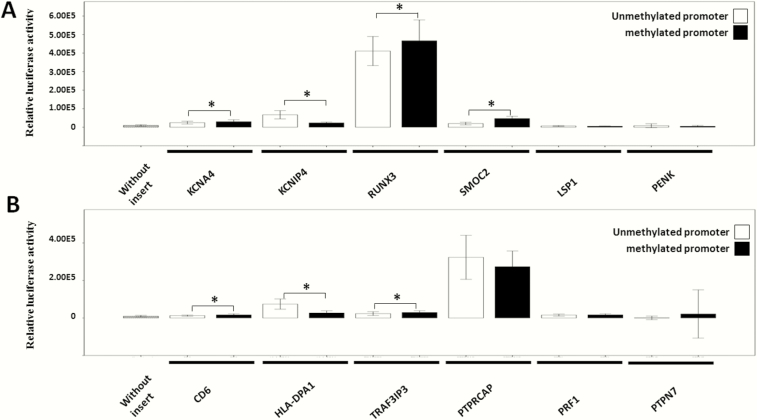
Effect of vector backbone CpG methylation on a CpG-free promoter by reporter gene analysis in transient transfections of the AC16 cell line. *A* and *B*, Gray bar represents reporter gene expression in the absence of promoter insert. White bars represent vector with unmethylated insert. Black bars represent vector with methylated insert (± 2 standard deviations). Experimental points were done in quadruplicate and experiments were repeated 4 times. For statistical analysis, a cutoff value of .004 was used. *The difference is statistically significant (the *P* value is under the cutoff value).

Among the 23 immune- or heart-related genes, 12 possessed several differentially methylated CpG sites in their promoter region (Supplementary Table 3). We performed a luciferase promoter reporter assay of methylated or unmethylated promoter regions. The cloned promoter sequence of each gene (including differentially methylated CpG sites found in CCC myocardium) was inserted into a CpG free-basic plasmid; unmethylated plasmids were subsequently methylated, and unmethylated or methylated plasmids were transfected as described. The promoter sequences are shown in Supplementary Table 1. In HEK293 cells, in vitro methylation of 5 plasmids induced strong reduction in promoter activity, while it caused increased promoter activity in 4 constructs (Figure 6 and Supplementary Table 8). In AC16 cells, in vitro methylation caused reduced promoter activity in 2 plasmids, and increased promoter activity in 4 plasmids (Figure 7 and Supplementary Table 8).

In silico analysis of transcription factor binding sites in the 40 differentially methylated promoter region CpG sites of the 12 studied genes (http://www.gene-regulation.com/pub/programs/alibaba2/index.html) was performed with a minimum matrix conservation of 75%. Promoter CpG sites overlapped with multiple predicted transcription factor binding regions. Specifically, we found that 21 of the 40 promoter CpG sites in 10 genes overlap binding sites for the Sp1 transcription factor (*CD6*cg13014558; *HLA-DPA1/HLA-DPB1*cg01132696; *HLA-DPA1/HLA-DPB1*cg03636880; *HLA-DPA1/HLA-DPB1*cg12893780; *HLA-DPA1/HLA-DPB1*cg20223237; *KCNA4*cg03506489; *KCNA4*cg05756220; *KCNA4*cg10387551; *KCNA4*cg22685409; *KCNIP4*cg00688962; *LSP1*cg05305434; *LSP1*cg07237979; *LSP1*cg19280572; *PRF1*cg19880751; *PRF1*cg23364656; *PTPN7*cg12436568; *PTPRCAP*cg12044599; *PTPRCAP*cg16408081; *PTPRCAP*cg17690322; *RUNX3*cg19774846; *SMOC2*cg10176110).

## DISCUSSION

We investigated for the first time the impact of cardiac DNA methylation on gene expression in the myocardium of patients with end-stage CCC. We actually state that 65% of the differentially methylated sites are overmethylated. Global methylation is similar in both groups (Figure 1). Methylation analysis was never done before either on CCC patients or in Chagas animal model. DNA methylation is one of the most studied epigenetic modifications. While DNA methylation in promoters is closely linked to downstream gene repression by affecting the affinity of transcription factors for their binding sites, gene body methylation has never been clearly associated with increased gene expression. This may explain why we detected direct or inverse correlations between our expression level and methylation level even though 62% of the differentially methylated genes were overmethylated.

This methylation pattern in complex tissues may be a reflex of the combination of its constituent cells (in our case cardiomyocytes, fibroblasts, endothelial cells, inflammatory cells) [19]. Reporter gene analyses disclosed that promoter methylation substantially modulated messenger RNA (mRNA) expression. In silico analysis of transcription factor binding indicated that promoter CpG sites overlapped multiple transcription factor binding regions in the studied genes, especially the Sp1 transcription factor. In 11 of the 12 studied genes, methylation was inversely associated with gene expression, either in tissue or reporter gene analyses.

The genes encoding *CD6, PRF1* [20], *PTPN6, PTPN7* [21], *HLA-DPA1* and *HLA-DPB1* [22, 23], *TRAF3IP3* [24], *PTPRCAP*, and *LSP1* are expressed in immune cells, which may explain their increased expression in CCC myocardium, as a result of the significant inflammatory infiltration—cells that are virtually absent in control heart tissue. In addition, our approach helped identify novel potential disease pathways and therapeutic targets in CCC. Indeed, 2 differentially expressed/differentially methylated genes were associated to regulation of potassium channels. *KCNA4*, upregulated in CCC, encodes the potassium voltage-gated channel Kv1.4, and *KCNIP4*, also upregulated in CCC, is a potassium channel–interacting protein that regulates Kv4.3 potassium channel function. Both modulate the duration and shape of the cardiac action potential. Potassium channel gene expression [14] and function [25, 26] were found to be disturbed in hearts of *T. cruzi*–infected mice. Interestingly, pharmacological or genetic modulation of potassium channels *Kcna4/Kv4.3* can cause or rescue severe arrhythmias similar to those found in CCC [27–31]. This suggests that methylation may be responsible for regulation of ion channels potentially involved in life-threatening ventricular arrhythmias found in CCC. The protein encoded by the *SMOC2* gene, upregulated in CCC, is highly expressed during wound healing and matrix assembly and remodeling, processes associated with fibrosis in CCC [32]; Transgenic mice overexpressing or underexpressing the *Smoc2* gene displayed increased /decreased kidney fibrosis in a kidney damage model, by modulating fibroblast proliferation and extracellular matrix deposition [33].

*PENK* mRNA, upregulated in CCC, is expressed mainly in the brain and heart and encodes preproenkephalin; enkephalin is a ligand of δ opioid receptors important in pain processing. δ opioid receptors are present in myocardium and immune cells; opioid signaling induces heart protection against ischemic injury in experimental models, but PENK is also known to be cardiodepressive in humans; increased levels of circulating PENK have been reported in heart failure and PENK is also an independent predictor of mortality after acute heart failure [34].

In addition, PENK drives T cells toward a Th1 profile and activates macrophages [35, 36]. Finally, *RUNX3*, upregulated in CCC, belongs to the Runt family of transcription factors. Runx3 interacts with transcription factor T-bet to induce maximal IFN-γ production in T cells [37] which are abundant in CCC myocardium [5]. This may indicate an additional driving factor for the potent, unrestricted activation of pathogenic IFN-γ producing T cells in CCC myocardium. The combined increase of *PENK* and *RUNX3* may indicate a novel immunoregulatory circuit for pathogenesis of CCC, where the increased local production of enkephalin can be an additional driver of the local activation of *RUNX3*-expressing Th1 CD4^+^ T cells and macrophages.

Significantly, the target genes modulated by DNA methylation in our study of CCC myocardium were distinct from those identified in dilated cardiomyopathy in previous studies [16, 17], suggesting that environmental/disease-specific factors regulating cardiac DNA methylation are different in the 2 cardiomyopathies, which may partially explain the worse prognosis observed in CCC. While our analysis started from differentially expressed genes that were also differentially methylated in the total gene body in CCC myocardial tissue as compared to controls, our reporter gene assays may have underestimated methylation effects in CpGs contained in other regions of the promoters, as well as methylation occurring in other parts of the gene body; on the other hand, methylase treatment of promoter sequences in plasmids also methylates CpG sites that were not found to be differentially methylated in vivo, which could also distinctly change expression patterns in a cell type-specific manner.

In silico analysis has shown that Sp1 is a major transcription factor for differentially methylated CpG regions [38]. In our set of Sp1 binding genes, promoter methylation was inversely correlated with expression, suggesting that DNA methylation may affect Sp1 binding and its transcriptional response both in heart cells and heart-infiltrating inflammatory cells. The Sp1 DNA binding properties and regulatory functions are different, depending on the promoter sequence and cellular background [39] and Sp1 may be a therapeutic target for restoring gene expression patterns [40].

## CONCLUSIONS

Our results suggest that DNA methylation modulates expression of pathogenetically relevant myocardium and immune system genes in CCC, especially those involving Th1 T cell activation, ion channels, myocardial remodeling, and protection. In-depth analysis of differentially methylated genes may help decipher the pathogenic mechanisms and provide therapeutic targets. Moreover, it will be essential to characterize the role of these epigenetic factors in CCC pathogenesis and progression to severe forms.

## Supplementary Data

Supplementary materials are available at *Clinical Infectious Diseases* online. Consisting of data provided by the authors to benefit the reader, the posted materials are not copyedited and are the sole responsibility of the authors, so questions or comments should be addressed to the corresponding author.

## Supplementary Material

Supplementary_table_1_20170516

Supplementary_table_2_20170516

Supplementary_table_3_20170516

Supplementary_table_4_20170516

Supplementary_table_5_20170516

Supplementary_table_6_20170516

Supplementary_table_7_20170516

Supplementary_table_8_20170516

## Notes

***Acknowledgments.*** The authors thank all the patients involved in this study; Elodie Bertosio, Flavie Sicard, and Olivier Zelphati for their technical expertise and their helpful assistance during the transfection experiments; and Marc Bartoli, Francesca Puppo, Albertina De Sario, and Milena Magalhaes for their help.

***Financial support.*** This work was supported by the Institut National de la Santé et de la Recherche Médicale (INSERM); Aix-Marseille University (Direction des Relations Internationales); the USP-COFECUB program; the ARCUS II PACA Brésil program; CNPq (the Brazilian National Council for Scientific and Technological Development); and FAPESP (São Paulo State Research Funding Agency-Brazil) (grant 2013/50302-3). This work is supported by the plan Laboratoire d’Excellence (LabEx) ParaFrap ANR-11-LABX-0024 . E. C.-N. and C. C. were recipients for an international program (Br-Fr-Chagas) funded by the French (Agence Nationale de Recherche) and Brazilian (FAPESP) science funding agencies. A. F. F. was a recipient of fellowships from FAPESP. E. C.-N. and J. K. are recipients of CNPq productivity awards.

***Potential conflicts of interest.*** All authors: No reported conflicts of interest. All authors have submitted the ICMJE Form for Disclosure of Potential Conflicts of Interest. Conflicts that the editors consider relevant to the content of the manuscript have been disclosed.
