# Supplementary material for: Whole-Genome Cardiac DNA Methylation Fingerprint and Gene Expression Analysis Provide New Insights in the Pathogenesis of Chronic Chagas Disease Cardiomyopathy
Source: Clin Infect Dis. 2017 May 30;65(7):1103–11. doi: 10.1093/cid/cix506 (PMC5849099; doi:10.1093/cid/cix506)
Supplement: Supplementary_table_1_20170516 [file cix506_suppl_supplementary_table_1_20170516.docx]

**Supplementary table 1:** Promoter sequences cloned in the pCpG‐free-basic-Lucia vector.

| **Gene** | **Insert (bp)** | **Sequence** |
| --- | --- | --- |
| ***RUNX3 (3)*** | **690** | CCTAGGAGCCCCCAGCCCTGCAGAGGGCCCCCCAGTGCGGGCAGGCCACCAGGTTTCCACAGGAAGGCCCTTCTCGGTGGGCAGGCCG[cg19774846]AGGCCAGGCGTGAGTGCTGGCAATGCCACTTCATGTGTTTCCCGTTGTACTTAGTCCCAACTCCCATAAAAGCCCCAGAGGTGCCACCCAGCCACACATGTGGACACCTCTCCCTGGCAACAATGGTGGTGGACAATGGCAGGGAGTCAGCCGCAGCCCAGGGGCGAGGTGGCATGGCAGGGAGCTCTCCGGCCGCTGGTGGATCCG[cg12459932]GGCTCTGGGCACTCGGTGAGGGGCCCGCGGGGCTCCTAGCCCGCCCAGGCCAATGCTGGCCTTAATTAAGAAGGAGTCTCCCACCCAGGAGCCAAACCACCCTCCTGGCCACGCCCACTGCAACCGCTTTCAGTTCTGTTTCTTGGGCCGCGTGCTGAGGCCAGCCTCACAAATAAAAGCCATCACTTTTCTATTTCTCTCTCTCTCTCTCTTTCTTTTTTTTTTTTTTCTTTTCCAAAAAGAGAGGCAGCCACAAGATCTTCTAAAAGGCCGTGACATCACGGCCCAGGTGACCGCGGCCCAGCCAATGAGCCAAGGCCGCGAGCAGGCTTCTCGCATCCTGTGAGCTGAGGTTGGGTTGACACTGGGAAGGCCTGGTCCCTCAACCAACTAGT |
| ***KCNA4 (2)*** | **239** | CCTAGGTTCCGGCCCCAACG[cg03506489]GGGACACTTCTCCAGGATTTGCTCCTCGCCTGTGGGGACCACAGCCAAAGACTCCTGGAATCGATCCCAGATCCTGGGAGTCGGGGGGCCCAGCCG[cg22685409]GGGTGCG[cg05756220]GGGAGTCG[cg15310492]CG[cg08490115]GCG[cg15044957]TCGTGACTGCACCCGCCCCGCCGCGCCGGGAGGCGCCCCTGGCGACCGTCGCG[cg17714025]AACG[cg10387551]CCACACCCTCGGTTCGGCCCTGGCCCCGCCCCCGCCCAGAACTAGT |
| ***CD6 (1)*** | **282** | CCTAGGGGACCGGGACAGGCAATGGGTCATTCACTCCCTCTTCCAATATGTACTGATTGAGCAGCTACTACATTATCAATTTTTTCTTCTGGGCCACCAGAAATGAGGCATGGCTCTTCCACTTCCCTTCTTCTTAGAAGACCCTCG[cg13014558]GGGAAGTGTGTCTGCATGGATGCG[cg21939215]TGTGCATGCG[cg27284288]TGTGTCTGGCTCCG[cg26427109]TGACTCTTGGGGTGCAGCCTGGATGGGTGGGCTCCCAGCACAGGCAGCTGGGGCTCTCCCCACCAGCCCCTGTAACAGCAGACTAGT |
| ***HLA-DPA1 (1)*** | **260** | CCTAGGGTCCCTTAGGCCAACCCGGCTGCTCCTGCGCCCTGGGCACGGGCCCGCG[cg12893780]GGGCTGCCCTGGGACCG[cg20223237]CCGGCCCAAAGCCCTCACTCACCTCGGCGCTGCAGGGTCATGGGCCCG[cg03636880]CCCAGCTCGTAGTTGTGTCTGCACATCCTGTCCGGCACTGCCCGCTTCTCCTCCAGGATGTCCTTCTGGCTGTTCCAGTACTCCGCAGCAGGCCGCCCCAGCTCCGTCACCGCCCGGAACTCCCCCACG[cg01132696]TCG[cg19990651]CTACTAGT |
| ***PTPRCAP (1)*** | **698** | ATGCATGAGTGCAAGCCTGGAGGCCAGGGGAGGGATGAGGGGACCCTGATGGGGCCTCCTAACTGATCCTGACCTCCCCG[CG17690322]GCCCCCTGCCCAGTCG[cg02740606]GACCTCTTCCAGGATGATCTGTACCCCGACACAGCCGGGCCCGAGGCAGCCCTGGAGGCTGAGGAGTGGGTGAGCGGGCGGGATGCCGACCCGATCCTCATCTCACTGCG[cg12044599]GGAGGCCTACGTGCCCAGCAAGCAGCGGGACCTGAAGATCAGCCG[cg23468927]GCGCAACGTGTTGTCTGACAGCCGGCCCGCCATGGCCCCGGGCTCCTCCCACCTAGGGGCCCCCGCCTCCACCACCACTGCTGCTGATGCCACCCCCAGCGGCAGCCTGGCCAGAGCCGGGGTATGCACGCGCAGGCAGGGGTGGTGCTGGGAGCCCCAGGTGTGGCCCTGGCCTGCTGTGTGGGCTTCAGCCATTCGCTGTCCCTCTCTGGGGTAGTGGTGGGTTTGGGAGGGCTATGGCTTAGCAGCCCCTGACCACCGCCCCCTACCCCTGCTTCATGTCCCCACAGGAGGCTGGGAAGCTGGAGGAGGTGATGCAGGAGCTGCGGGCCCTGAGGGCGCTGGTCAAGGAGCAGGGCGACCGCATCTGCCGCCTGGAGGAGCAGCTGGGCCG[cg16408081]CATGGAGAACGGGGATGCGTAGGGCCACAGCCACACGCCACCTTCATAAGCTT |
| ***TRAF3IP3 (1)*** | **593** | CCTAGGCCTCTCCACTATTTTAGCCTGAACTTCTTGGGAATTTCCTGGCTGCATCATTTGTGATGGCTTCATCTTAATGCATGCAAATCAGTTCCCTTTAGTTTTTCCCTAAATTTCCTAGTCCG[cg08655071]GTTGACCAGATAAAAAAATGTGTAGAAGACTCTGCCTTCTATTCCTTCCTTCATCCCCCAGCTACCCACCTTTACACTCCCTCCTCATTGCTCCAGAAAGGAGCAAATCCAGAGTTGGTCTCATTCCAGAATCTGAGAAAGACACCACCTACTTCAGAAGAGCAGAAACTCTTTACAACACCAGGATTGGGAAGAGGTGGCTAAAGAAAAAGGAAAATCCTTACACAAAGAACTACCTGATACACATGAAAGCCTACACTGAGAACATCTCATCTAGGTCTTACCACCAAAAAGGCATTTTTTTCCCTCTCATAGAAACCACAAAGGTCTGAATCTGACCCAGAGGACTATCCTTTTCTAATCATGGAAAGGAATCTTGACTTGAAACTGTCCAAGAGCTCCGACCAATCCAGTAGCAAGAGAAAGGGTGGCCGGGGTGCCTACTGATGGGAGGAGCTCCTGACTAGT |
| ***PENK (2)*** | **262** | CCTAGGTGCCCCGGCCGGGAGGCAGGCGGTGCTCGGGTGCAGCTTCGGGGCTAATCCGAGGGCTGCGTGTCAAGCCCGCACATTGAGGCCTGCG[cg16072688]GAGAACTGAGACCCCAGTCCCCGCAAGCCCAGCCTACGCAGGAGAGCGCTGCCCTCTAGCGACTATAATGGGACATGCAGCGCGGCCGGAGCCCCGCGGGAGCAGCGCCGCTGCAGGTCCTAGCGACTGTAGAAATCAGCCCTTTGCAGAGGGCGCAGAGGGACTAGT |
| ***PRF1 (2)*** | **413** | CCTAGGGAGGCACAGTGAGGCTGAAGAACCCTACCAGTCCACACTGCTGGTGCATAACCGAGCTGCCCAAGCCCCGGCGGTCTGGCG[cg15293582]TGTAGGCCCATGCTCTGAGCCGCCGCCTCTGCTTGCCTCTTACATCCCACACATGCG[cg02374486]ATGCTGTGCATCAGAAGCAAGGAGATGGCCCTGCTGGCCTGTTCATCAACACCAGGGCCGAGTCTCAAAGTCCTCAGCGCCCCGCCCTCCTCCG[cg23364656]CCTGTGTGCCCTGAGTCCCCG[cg23059461]AGCCCCAGCAGCTCTACTCGGCAGATGAGCCTCTGGCCCTGCTGCTCGCTTCCTGAGGGCTGTCAGTGGGGAGCCG[cg19880751]GATGAGGGCTGAGGACAGGGTGGGTGCTTGTGGGAGGGGAGAGCACAAAGGACCTGTGACCACAGCTGGGGGACTAGT |
| ***SMOC2 (3)*** | **245** | CCTAGGGTGTGCACGTGTGTGTGTGAGAGTGCGCGCGGGGAAGGAGGCACAGAGACAGCCCGGACAGGCCACTGCG[cg10176110]CAGCCCTGGTGGCCCCCGCTCCACCTCTCGCTCCGCAGACCCGCGCCAGGGAGGCCTCTGGGCCGCAGCGGGCACCGGAGCGGAGCGGGCGCGGCAGCGGGCGCTGGGAGGTGGGGCTGGGGGAGGAGAGGGGGAGGGAGAGAGGCGGGCGGGAGGGGAGGATACTAGT |
| ***PTPN7 (2)*** | **527** | ATGCATACACTGACTGGTGTGGGGGGTGAGGCAGAGGCCAGTCTCCAGGTCACTGCTCG[cg15027815]CAAGCATGTTAGGGATTCCCAGGTTCCTGCTTCCCTCCAGTCTGTCCTGAGCACGGGAACCAACCCCTTTCCCAGCCCTAATCGGCCTCCTCCTCAGGGAAACCCACCCACTCCGGAGAGGAACGCAGGCTGCAGGGAAGGAGGTGCCAGGATCAGCTGGCCATGGAGGGGCTCAAAAAAGAGCCAGCCCCTGTCCCCACTGCCAAGAGCAAGGGCGGGTGCTGGGAGGCCTTCTGAGCCTCTGGGGGCTGTTCTTGTTCTTCG[cg23506842]TCTCAACCCTCCTCCTCAAGGGGCCAGCAGAAGGCCAGCCCCCTCCCGACCACCCCCTCCGGCATGCCTACCCCCTGTGCCTAGGGTACCTCATTTCTTTTTTGCTGTCACCATTTGACAGCTGAATCAGGGAAGTGGGAGGAAGAAAGAGGACGTGGATTGTGCATGTTGGGGGCCGGGAAGGGAAGGGCG[cg12436568]TTTGAAAAGCTT |
| ***KCNIP4 (1)*** | **408** | CCTAGGAGCCCCTCCCCAGAGCAGGCGCTGCTCGCAGTGACAAGGGGTCCTAGAGGCGCCCGCTGAGAGCCG[cg18347642]CTGGCAACTCCCGGGGGTGTCCTGCCCAGCCCGAAGTCACCTCGGGTCTCTTTCGGGTCCATCTACTCTGTCGCCAGAGGCACCGTGCCACCCGCGGCGCTTTCCTCTCCCCGCACCCCGCCCCCCGCATCCCCAGTCCGGTGACTTGGCAGCAGGTGGCAGGGGCGGCGCTCACGAGGGCGAGGGGCTGGGGTCCCCG[cg00688962]CACTCGAGCCGCCCGTGCAGCCCGCCCGCTTCCAGCCGCCGGCGCGAAGCTGCCGGCGCCTCGGCCTCCCCCGCCACCCCGCGCGCCCCCGCCGCCAAGGGCCGCGCAGCGCCCCCGGCGCCCCCCGCCGGACTAGT |
| ***LSP1 (2)*** | **361** | CCTAGGTTCGAGAAAAGACTCCACCAAGTTCCATGAGCACCTGTAGGAGAGCCG[cg19280572]GCCTCTGGGTAGATTTCACCCAAAGAAGCTAAAACGCTCACGTGGTGCTTATGGGATCACAGACGTGTTTGTCCCTGATGCTGCAGGAGAACTGGCCACTCACAGGTGGACCTGATGGCCAGAGCTACTAGCG[cg07237979]GGGACCAGGGGTAACATGGGTCCCCCTCCCCG[cg05305434]AGCCATGAAGAGCTGCCTGCGGCCATCTTGGCCCTCGCACCCCGTCTCTGTCACCCCAGGCCCCTGTAACTTGCTTAACGCTTCCTGAGGGGTGGTTTGGGTTTTTTTTTTTTTCTTCCTCTCCTCTTGTAGCAACACTAGT |

Cloning sites are underlined

Service providers: (1) GeneCust (Luxembourg, Luxembourg); (2) Life Technologies/GeneArt (Sain Aubin, France); (3) Sigma/GeneWiz (Saint-Quentin Fallavier, France).
