# Supplementary material for: Whole-Genome Cardiac DNA Methylation Fingerprint and Gene Expression Analysis Provide New Insights in the Pathogenesis of Chronic Chagas Disease Cardiomyopathy
Source: Clin Infect Dis. 2017 May 30;65(7):1103–11. doi: 10.1093/cid/cix506 (PMC5849099; doi:10.1093/cid/cix506)
Supplement: Supplementary_table_2_20170516 [file cix506_suppl_supplementary_table_2_20170516.docx]

**Supplementary table 2:** Gene expression analysis were validated by gene specific quantitative real-time PCR. This table provided information on the probes and housekeeping genes used.

| **Genes** | **TaqMan assays** | **Expression Array** | | **Gene specific qRTPCR** | |
| --- | --- | --- | --- | --- | --- |
|  |  | **Fold change** | **P value** | **Fold change** | **P value** |
| *ABRA* | Hs00611714_s1 | -4.34 | 3.00E-02 | -8.23 | 1.90E-01 |
| ***ADRB1*** | Hs02330048_s1 | -2.21 | 1.00E-02 | -2.13 | 1.00E-02 |
| ***AIF1*** | hs00741549_g1 | 4.25 | 2.00E-02 | 7. 91 | 3.00E-03 |
| ***ANGPT1*** | Hs00181613_m1 | 4.04 | 2.00E-03 | 3.03 | 5.00E-03 |
| ***APOA1*** | Hs00163641_m1 | 4.56 | 2.00E-02 | 3.93 | 2.00E-03 |
| ***ATP2A2*** | Hs00854939_g1 | -3.34 | 6.00E-04 | -3.52 | 8.00E-05 |
| ***ATP2A3*** | Hs01024559_mH | 3.07 | 7.00E-03 | 3.28 | 4.00E-03 |
| ***B4GALT6*** | Hs00191135_m1 | -2.11 | 2.00E-02 | -2.12 | 6.00E-05 |
| ***C1QC*** | hs00757779_m1 | 3.33 | 1.00E-02 | 5. 75 | 9.00E-03 |
| ***CAMK4*** | Hs00174318_m1 | 6.16 | 6.00E-04 | 28.15 | 1.00E-03 |
| ***CASP8*** | hs01018156_m1 | 6.2 | 3.27E-04 | 6. 78 | 4.00E-04 |
| ***CCL5*** | hs00174575_m1 | 77.89 | 3.17E-05 | 65. 16 | 1.00E-03 |
| ***CD74*** | hs00959498_g1 | 3.16 | 2.00E-02 | 5. 72 | 2.00E-03 |
| *CDC42* | Hs00854939_g1 | -2.98 | 2.00E-02 | -1.06 | 7.20E-01 |
| ***CNN1*** | hs00154543_m1 | -4.71 | 2.00E-02 | -5. 25 | 4.50E-02 |
| ***COL1A2*** | Hs01028971_m1 | 2.88 | 2.00E-02 | 2.93 | 2.00E-02 |
| ***COL3A1*** | Hs00943809_m1 | 3.54 | 9.00E-03 | 3.46 | 2.00E-03 |
| *ESRRA* | Hs01067166_g1 | -2.2 | 1.00E-03 | 2.32 | 3.30E-01 |
| ***F2RL2*** | hs00187982_m1 | 5.19 | 2.00E-02 | 8. 74 | 6.70E-02 |
| ***GATA4*** | Hs01034629_m1 | -2.11 | 2.00E-02 | -2.52 | 3.00E-02 |
| *GPD1* | hs01100039_m1 | -3.028 | 3.00E-02 | 1. 13 | 9.60E-01 |
| ***HOOK1*** | hs00210665_m1 | -4.28 | 2.00E-02 | -2. 71 | 1.30E-02 |
| *HOPX* | hs00261238_m1 | -5.48 | 1.50E-02 | -3. 55 | 1.22E-01 |
| ***IL18*** | Hs00155517_m1 | 4.81 | 1.00E-02 | 6.52 | 6.00E-03 |
| ***LEF1*** | hs01547250_m1 | 9.15 | 6.00E-03 | 10. 55 | 3.00E-03 |
| ***MYH6*** | Hs00854939_g1 | -4.92 | 2.00E-03 | -38.63 | 4.00E-02 |
| ***NCF1*** | hs00165362_m1 | 14.69 | 2.00E-03 | 19. 79 | 1.00E-03 |
| ***NCF2*** | hs01084940_m1 | 3.55 | 2.00E-02 | 6. 23 | 6.00E-03 |
| *NFATC2* | hs00905451_m1 | 3.04 | 2.00E-02 | 1. 97 | 2.00E-01 |
| *NPPA* | Hs00383230_g1 | -2.12 | 3.00E-03 | 8.66 | 1.20E-01 |
| ***P2RY2*** | hs01923024_s1 | -3.56 | 9.00E-03 | -2. 76 | 1.00E-02 |
| ***PIK3CG*** | Hs00277090_m1 | 5.37 | 1.00E-03 | 4.93 | 1.00E-03 |
| ***PIM1*** | Hs01065498_m1 | -2.72 | 4.00E-02 | -4.27 | 6.00E-02 |
| ***POSTN*** | Hs01566734_m1 | 6.29 | 3.00E-02 | 8.32 | 4.00E-02 |
| ***PPP2R2B*** | Hs00270227_m1 | 9.74 | 2.00E-03 | 9.96 | 1.00E-03 |
| ***PRKCB*** | Hs01030676_m1 | 7.02 | 4.00E-04 | 7.86 | 1.00E-02 |
| ***PRKCQ*** | hs00234704_m1 | 16.8 | 1.05E-05 | 25. 09 | 5.00E-05 |
| ***RASL10B*** | Hs00430966_m1 | -2.19 | 2.00E-02 | -3.16 | 6.00E-03 |
| ***RLN2*** | hs00754884_s1 | 3.77 | 1.00E-02 | 5. 27 | 1.00E-04 |
| ***RRAD*** | Hs00188163_m1 | -7.91 | 1.00E-02 | -11.48 | 5.00E-02 |
| ***SOCS1*** | hs00705164_s1 | 3.93 | 2.00E-03 | 4. 89 | 8.00E-03 |
| ***STAT1*** | hs01014002_m1 | 3.87 | 2.00E-02 | 6. 76 | 2.00E-02 |
| *TGFBR2* | hs00559661_m1 | 3.28 | 5.00E-02 | 1. 33 | 4.70E-01 |
| ***TNFAIP3*** | Hs01568118_m1 | 4.49 | 1.00E-02 | 3.96 | 5.00E-03 |

Heart tissue samples (20-30 mg) were crushed with ceramic beads (CK14, diameter 1,4 mm) in 350 µl of lysis buffer RLT supplemented with 3.5 µl of β-mercapto-ethanol. The Precellys-24 device was used. Total RNA was extracted from biopsies using the RNeasy Mini Kit adapted with Trizol. RNA quantity and quality were measured with a NanoVue Plus Spectrophotometer and a 2100 bioanalyser using RNA 6000 Nano Kit. The RNA Integrity Number was calculated and all RNAs with a RIN above 7 were selected for gene expression analyses.

Whole human genome expression analysis was done on **SurePrint G3 Human Gene Expression v1 8x60K** arrays. Microarrays were done using the Low Input Quick Amp Labeling One-Color Kit and the One-color RNA Spike-in Kit following the manufacturer's standard protocol (Agilent One-Color Microarray-Based Gene Expression Analysis Low Input Quick Amp Labeling). 100 ng of total RNA input was used. After cDNA synthesis. amplification, labeling and purification, the reaction efficacy was checked using the NanoDrop. The hybridization was performed for 17 hours at 10 rpm and 65°C in a Hybridization Oven. Slide Wash was done according to the manufacturer's recommendation. Cy3 intensities were detected by one-color scanning using Agilent DNA microarray scanner at 3 micron resolution. Raw data from scanned image files were extracted using Feature Extraction software (10.7.3.1). Spike-in used in the experiment gave reliable data and all arrays passed the quality control. The microarrays were therefore analyzed using GeneSpring software (11.5.1).

For gene expression analysis, by single probe quantitative RT-PCR, 400 ng of the total RNA was reverse transcribed to cDNA by using High capacity cDNA archive kit 2X from Applied Biosystems. The reactions were carried out on an ABI 7900 real time PCR thermocycler according to the manufacturer’s recommendations. Expression levels were reported as 2^-DCT^ values.

Genes in bold were significantly differentially expressed on array and by qRTPCR.

Housekeeping genes: 18S (hs03003631_g1) and RPLP0 (hs99999902_m1).
