# Supplementary material for: Whole-Genome Cardiac DNA Methylation Fingerprint and Gene Expression Analysis Provide New Insights in the Pathogenesis of Chronic Chagas Disease Cardiomyopathy
Source: Clin Infect Dis. 2017 May 30;65(7):1103–11. doi: 10.1093/cid/cix506 (PMC5849099; doi:10.1093/cid/cix506)
Supplement: Supplementary_table_3_20170516 [file cix506_suppl_supplementary_table_3_20170516.docx]

**Supplementary table 3:** List of the genes differentially expressed and differentially methylated on Chagas heart tissue biopsies and healthy donors.

| **Gene symbol** | **UniGene ID** | **Gene Name** | **Differentially methylated CpGs in the gene unit (1)** | **Differentially methylated CpGs in the 10kb proximal promoter (2)** |
| --- | --- | --- | --- | --- |
| ***ABCC3*** | Hs.463421 | ATP-binding cassette, sub-family C (CFTR/MRP), member 3 | 1/28 | 1/26 |
| ***ABLIM2*** | Hs.233404 | actin binding LIM protein family, member 2 | 1/133 | 1/130 |
| ***ACAP1*** | Hs.337242 | ArfGAP with coiled-coil, ankyrin repeat and PH domains 1 | 3/46 | 3/30 |
| ***ADAM8*** | Hs.501574 | ADAM metallopeptidase domain 8 | 1/66 | 1/57 |
| ***ADARB2*** | Hs.657984 | adenosine deaminase, RNA-specific, B2 | 1/377 | 1/364 |
| ***ADCY7*** | Hs.513578 | adenylate cyclase 7 | 1/31 | 1/30 |
| ***ADORA3*** | Hs.281342 | adenosine A3 receptor | 4/25 | 4/21 |
| ***ADRB1*** | Hs.99913 | adrenergic, beta-1-, receptor | 2/10 | 2/10 |
| ***AGAP3*** | Hs.647075 | ArfGAP with GTPase domain, ankyrin repeat and PH domain 3 | 2/98 | 2/97 |
| ***AIM1*** | Hs.728842 | absent in melanoma 1 | 1/29 | 1/27 |
| ***AIM1L*** | Hs.128738 | absent in melanoma 1-like | 2/39 | 1/29 |
| ***AIM2*** | Hs.281898 | absent in melanoma 2 | 2/11 | 2/11 |
| ***ALOX15B*** | Hs.111256 | arachidonate 15-lipoxygenase, type B | 1/14 | 0/7 |
| ***AMICA1*** | Hs.16291 | adhesion molecule, interacts with CXADR antigen 1 | 2/20 | 2/20 |
| ***ANKRD36BP2*** | Hs.105323 | ankyrin repeat domain 36B pseudogene 2 | 1/13 | 1/13 |
| ***ANKRD44*** | Hs.432706 | ankyrin repeat domain 44 | 1/37 | 1/35 |
| ***APBA2*** | Hs.618112 | amyloid beta (A4) precursor protein-binding, family A, member 2 | 2/69 | 2/66 |
| ***APBB1IP*** | Hs.310421 | amyloid beta (A4) precursor protein-binding, family B, member 1 interacting protein | 4/23 | 4/22 |
| ***APCDD1L*** | Hs.119286 | adenomatosis polyposis coli down-regulated 1-like | 2/29 | 2/28 |
| ***APOBR*** | Hs.200333 | apolipoprotein B receptor | 2/36 | 2/27 |
| ***AQP10*** | Hs.259048 | aquaporin 10 | 1/30 | 1/10 |
| ***ARAP2*** | Hs.479451 | ArfGAP with RhoGAP domain, ankyrin repeat and PH domain 2 | 1/15 | 1/15 |
| ***ARHGAP30*** | Hs.389374 | Rho GTPase activating protein 30 | 2/42 | 2/14 |
| ***ARHGAP4*** | Hs.701324 | Rho GTPase activating protein 4 | 1/43 | 1/27 |
| ***ARHGAP9*** | Hs.437126 | Rho GTPase activating protein 9 | 3/35 | 3/31 |
| ***ARHGEF16*** | Hs.87435 | Rho guanine nucleotide exchange factor (GEF) 16 | 2/66 | 2/56 |
| ***ASCL2*** | Hs.152475 | achaete-scute complex homolog 2 (Drosophila) | 1/71 | 1/60 |
| ***ATP2A3*** | Hs.513870 | ATPase, Ca++ transporting, ubiquitous | 5/60 | 3/46 |
| ***ATP8A2*** | Hs.444957 | ATPase, aminophospholipid transporter, class I, type 8A, member 2 | 1/56 | 1/56 |
| ***ATP8B2*** | Hs.435700 | ATPase, class I, type 8B, member 2 | 1/34 | 1/31 |
| ***ATXN7L1*** | Hs.489603 | ataxin 7-like 1 | 2/74 | 2/74 |
| ***BATF*** | Hs.509964 | basic leucine zipper transcription factor, ATF-like | 3/21 | 3/19 |
| ***BCAR1*** | Hs.479747 | breast cancer anti-estrogen resistance 1 | 1/58 | 1/54 |
| ***BHLHE22*** | Hs.591870 | basic helix-loop-helix family, member e22 | 1/58 | 1/45 |
| ***BMP7*** | Hs.473163 | bone morphogenetic protein 7 | 4/40 | 4/40 |
| ***C10orf105*** | Hs.568788 | chromosome 10 open reading frame 105 | 1/29 | 1/28 |
| ***C14orf182*** | Hs.660867 | chromosome 14 open reading frame 182 | 1/18 | 1/18 |
| ***C16orf54*** | Hs.331095 | chromosome 16 open reading frame 54 | 5/7 | 3/7 |
| ***C1QTNF1*** | Hs.201398 | C1q and tumor necrosis factor related protein 1 | 1/41 | 1/40 |
| ***C1QTNF2*** | Hs.110062 | C1q and tumor necrosis factor related protein 2 | 1/16 | 1/16 |
| ***C1QTNF6*** | Hs.22011 | C1q and tumor necrosis factor related protein 6 | 2/29 | 1/27 |
| ***C1QTNF7*** | Hs.153714 | C1q and tumor necrosis factor related protein 7 | 1/30 | 1/29 |
| ***C1S*** | Hs.458355 | complement component 1, s subcomponent | 2/34 | 1/31 |
| ***C2*** | Hs.408903 | complement component 2 | 2/231 | 2/193 |
| ***C5orf20*** | Hs.152477 | chromosome 5 open reading frame 20 | 1/18 | 1/17 |
| ***C5orf56*** | Hs.658288 | chromosome 5 open reading frame 56 | 1/28 | 1/26 |
| ***C7orf63*** | Hs.657403 | chromosome 7 open reading frame 63 | 1/21 | 0/18 |
| ***C9orf3*** | Hs.434253 | chromosome 9 open reading frame 3 | 1/52 | 1/48 |
| ***CACNA1B*** | Hs.495522 | calcium channel, voltage-dependent, N type, alpha 1B subunit | 2/44 | 2/43 |
| ***CACNA2D3*** | Hs.656687 | calcium channel, voltage-dependent, alpha 2/delta subunit 3 | 1/82 | 1/82 |
| ***CCDC85C*** | Hs.709288 | coiled-coil domain containing 85C | 2/54 | 2/51 |
| ***CCDC88B*** | Hs.98564 | coiled-coil domain containing 88B | 2/52 | 2/39 |
| ***CCDC88C*** | Hs.525536 | coiled-coil domain containing 88C | 2/89 | 1/83 |
| ***CCL19*** | Hs.50002 | chemokine (C-C motif) ligand 19 | 1/7 | 1/6 |
| ***CCR7*** | Hs.370036 | chemokine (C-C motif) receptor 7 | 2/17 | 2/10 |
| ***CD160*** | Hs.488237 | CD160 molecule | 1/14 | 1/13 |
| ***CD247*** | Hs.156445 | CD247 molecule | 5/41 | 5/40 |
| ***CD300LF*** | Hs.567706 | CD300 molecule-like family member f | 1/8 | 1/8 |
| ***CD37*** | Hs.166556 | CD37 molecule | 3/24 | 3/24 |
| ***CD3D*** | Hs.504048 | CD3d molecule, delta (CD3-TCR complex) | 1/12 | 1/12 |
| ***CD3G*** | Hs.2259 | CD3g molecule, gamma (CD3-TCR complex) | 1/28 | 1/13 |
| ***CD48*** | Hs.243564 | CD48 molecule | 1/12 | 1/9 |
| ***CD5*** | Hs.58685 | CD5 molecule | 1/13 | 1/8 |
| ***CD52*** | Hs.276770 | CD52 molecule | 1/12 | 1/11 |
| ***CD6*** | Hs.729486 | CD6 molecule | 7/23 | 7/21 |
| ***CD74*** | Hs.436568 | CD74 molecule, major histocompatibility complex, class II invariant chain | 4/16 | 4/15 |
| ***CD8A*** | Hs.85258 | CD8a molecule | 1/45 | 1/44 |
| ***CD96*** | Hs.142023 | CD96 molecule | 1/39 | 1/24 |
| ***CDH9*** | Hs.272212 | cadherin 9, type 2 (T1-cadherin) | 1/15 | 1/15 |
| ***CDX1*** | Hs.1545 | caudal type homeobox 1 | 1/33 | 0/19 |
| ***CEBPE*** | Hs.558308 | CCAAT/enhancer binding protein (C/EBP), epsilon | 2/17 | 2/14 |
| ***CGNL1*** | Hs.148989 | cingulin-like 1 | 1/37 | 1/34 |
| ***CHDH*** | Hs.126688 | choline dehydrogenase | 1/27 | 1/22 |
| ***CHST15*** | Hs.287537 | carbohydrate (N-acetylgalactosamine 4-sulfate 6-O) sulfotransferase 15 | 1/57 | 1/56 |
| ***CLDN14*** | Hs.660278 | claudin 14 | 1/29 | 1/29 |
| ***CLNK*** | Hs.678910 | cytokine-dependent hematopoietic cell linker | 1/18 | 1/18 |
| ***CNN1*** | Hs.465929 | calponin 1, basic, smooth muscle | 3/47 | 3/32 |
| ***COL16A1*** | Hs.368921 | collagen, type XVI, alpha 1 | 3/38 | 3/23 |
| ***COL5A1*** | Hs.210283 | collagen, type V, alpha 1 | 2/79 | 2/79 |
| ***CORO1A*** | Hs.415067 | coronin, actin binding protein, 1A | 4/21 | 4/17 |
| ***CORO2A*** | Hs.113094 | coronin, actin binding protein, 2A | 1/13 | 0/8 |
| ***COTL1*** | Hs.289092 | coactosin-like 1 (Dictyostelium) | 1/29 | 1/27 |
| ***CPNE7*** | Hs.461775 | copine VII | 1/57 | 1/51 |
| ***CPXM2*** | Hs.656887 | carboxypeptidase X (M14 family), member 2 | 3/45 | 3/45 |
| ***CXCL16*** | Hs.730800 | chemokine (C-X-C motif) ligand 16 | 1/38 | 1/22 |
| ***CXCR6*** | Hs.34526 | chemokine (C-X-C motif) receptor 6 | 1/10 | 1/9 |
| ***CYTH4*** | Hs.170944 | cytohesin 4 | 1/17 | 0/10 |
| ***DAPP1*** | Hs.436271 | dual adaptor of phosphotyrosine and 3-phosphoinositides | 2/8 | 2/8 |
| ***DCHS2*** | Hs.655664 | dachsous 2 (Drosophila) | 2/34 | 2/34 |
| ***DDX31*** | Hs.660767 | DEAD (Asp-Glu-Ala-Asp) box polypeptide 31 | 1/30 | 1/16 |
| ***DEF6*** | Hs.15476 | differentially expressed in FDCP 6 homolog (mouse) | 1/19 | 1/19 |
| ***DENND1C*** | Hs.236449 | DENN/MADD domain containing 1C | 2/44 | 1/20 |
| ***DENND2D*** | Hs.557850 | DENN/MADD domain containing 2D | 4/27 | 4/25 |
| ***DLEC1*** | Hs.714499 | deleted in lung and esophageal cancer 1 | 2/22 | 2/21 |
| ***DLEU1*** | Hs.591229 | deleted in lymphocytic leukemia 1 (non-protein coding) | 1/98 | 1/98 |
| ***DNAJB5*** | Hs.237506 | DnaJ (Hsp40) homolog, subfamily B, member 5 | 1/7 | 1/7 |
| ***DNALI1*** | Hs.406050 | dynein, axonemal, light intermediate chain 1 | 4/31 | 4/30 |
| ***DNASE1L3*** | Hs.476453 | deoxyribonuclease I-like 3 | 2/8 | 2/7 |
| ***DOK2*** | Hs.71215 | docking protein 2, 56kDa | 2/25 | 2/24 |
| ***DOK3*** | Hs.720849 | docking protein 3 | 1/60 | 1/40 |
| ***DQX1*** | Hs.191705 | DEAQ box RNA-dependent ATPase 1 | 4/52 | 0/29 |
| ***DRD4*** | Hs.99922 | dopamine receptor D4 | 2/37 | 0/29 |
| ***DUSP13*** | Hs.178170 | dual specificity phosphatase 13 | 1/29 | 1/25 |
| ***EBI3*** | Hs.501452 | Epstein-Barr virus induced 3 | 1/24 | 1/10 |
| ***ERI2*** | Hs.248437 | ERI1 exoribonuclease family member 2 | 1/43 | 1/43 |
| ***F2R*** | Hs.482562 | coagulation factor II (thrombin) receptor | 2/27 | 2/26 |
| ***FAAH*** | Hs.720143 | fatty acid amide hydrolase | 1/18 | 1/17 |
| ***FAM65B*** | Hs.559459 | family with sequence similarity 65, member B | 4/64 | 4/64 |
| ***FAM78B*** | Hs.133029 | family with sequence similarity 78, member B | 1/31 | 1/31 |
| ***FAM83B*** | Hs.657974 | family with sequence similarity 83, member B | 2/14 | 2/14 |
| ***FANCA*** | Hs.290154 | Fanconi anemia, complementation group A | 2/34 | 2/28 |
| ***FASLG*** | Hs.2007 | Fas ligand (TNF superfamily, member 6) | 1/6 | 1/6 |
| ***FBLIM1*** | Hs.530101 | filamin binding LIM protein 1 | 1/26 | 1/26 |
| ***FCHO1*** | Hs.96485 | FCH domain only 1 | 5/40 | 5/29 |
| ***FCN1*** | Hs.440898 | ficolin (collagen/fibrinogen domain containing) 1 | 1/6 | 1/6 |
| ***FLT3LG*** | Hs.428 | fms-related tyrosine kinase 3 ligand | 2/60 | 0/24 |
| ***FMNL1*** | Hs.100217 | formin-like 1 | 2/39 | 2/31 |
| ***FNDC9*** | Hs.437066 | fibronectin type III domain containing 9 | 1/9 | 1/8 |
| ***FOLR2*** | Hs.433159 | folate receptor 2 (fetal) | 1/19 | 1/5 |
| ***FOXP2*** | Hs.282787 | forkhead box P2 | 1/43 | 1/43 |
| ***FRZB*** | Hs.128453 | frizzled-related protein | 1/16 | 1/16 |
| ***FURIN*** | Hs.513153 | furin (paired basic amino acid cleaving enzyme) | 1/42 | 0/24 |
| ***GALNT6*** | Hs.505575 | UDP-N-acetyl-alpha-D-galactosamine:polypeptide N-acetylgalactosaminyltransferase 6 (GalNAc-T6) | 1/26 | 1/23 |
| ***GATA4*** | Hs.243987 | GATA binding protein 4 | 2/118 | 2/104 |
| ***GFI1*** | Hs.73172 | growth factor independent 1 transcription repressor | 1/67 | 1/67 |
| ***GFRA2*** | Hs.441202 | GDNF family receptor alpha 2 | 1/40 | 1/40 |
| ***GGN*** | Hs.447948 | gametogenetin | 1/44 | 1/28 |
| ***GHRL*** | Hs.590080 | ghrelin/obestatin prepropeptide | 1/27 | 0/19 |
| ***GLIS1*** | Hs.306691 | GLIS family zinc finger 1 | 1/66 | 1/63 |
| ***GLIS2*** | Hs.592087 | GLIS family zinc finger 2 | 3/46 | 3/45 |
| ***GNMT*** | Hs.144914 | glycine N-methyltransferase | 2/42 | 2/39 |
| ***GPC6*** | Hs.444329 | glypican 6 | 2/73 | 2/73 |
| ***GPR160*** | Hs.231320 | G protein-coupled receptor 160 | 1/31 | 1/31 |
| ***GPX3*** | Hs.730658 | glutathione peroxidase 3 (plasma) | 1/18 | 1/17 |
| ***GRIK3*** | Hs.128848 | glutamate receptor, ionotropic, kainate 3 | 2/39 | 2/39 |
| ***GSDMB*** | Hs.306777 | gasdermin B | 1/28 | 1/28 |
| ***GTF2IRD1*** | Hs.647056 | GTF2I repeat domain containing 1 | 1/48 | 1/38 |
| ***HAAO*** | Hs.368805 | 3-hydroxyanthranilate 3,4-dioxygenase | 1/27 | 1/18 |
| ***HCP5*** | Hs.728757 | HLA complex P5 (non-protein coding) | 1/148 | 1/148 |
| ***HCST*** | Hs.117339 | hematopoietic cell signal transducer | 1/26 | 1/17 |
| ***HLA-DMA*** | Hs.728759 | major histocompatibility complex, class II, DM alpha | 2/217 | 1/194 |
| ***HLA-DMB*** | Hs.351279 | major histocompatibility complex, class II, DM beta | 1/63 | 1/58 |
| ***HLA-DOB*** | Hs.1802 | major histocompatibility complex, class II, DO beta | 1/36 | 1/34 |
| ***HLA-DPA1*** | Hs.347270 | major histocompatibility complex, class II, DP alpha 1 | 11/107 | 11/106 |
| ***HLA-DPB1*** | Hs.485130 | major histocompatibility complex, class II, DP beta 1 | 11/102 | 11/102 |
| ***HLA-F*** | Hs.519972 | major histocompatibility complex, class I, F | 1/125 | 0/87 |
| ***HLA-J*** | Hs.720762 | major histocompatibility complex, class I, J (pseudogene) | 1/103 | 1/68 |
| ***HMHA1*** | Hs.465521 | histocompatibility (minor) HA-1 | 3/60 | 3/43 |
| ***HOOK1*** | Hs.378836 | hook homolog 1 (Drosophila) | 1/15 | 1/15 |
| ***ICAM4*** | Hs.706750 | intercellular adhesion molecule 4 (Landsteiner-Wiener blood group) | 1/42 | 1/13 |
| ***IGSF21*** | Hs.212511 | immunoglobin superfamily, member 21 | 3/71 | 3/70 |
| ***IGSF22*** | Hs.434152 | immunoglobulin superfamily, member 22 | 1/39 | 0/23 |
| ***IKZF3*** | Hs.444388 | IKAROS family zinc finger 3 (Aiolos) | 1/20 | 0/18 |
| ***IL12RB1*** | Hs.567294 | interleukin 12 receptor, beta 1 | 1/22 | 1/22 |
| ***IL16*** | Hs.459095 | interleukin 16 | 1/33 | 1/31 |
| ***IL17RB*** | Hs.654970 | interleukin 17 receptor B | 1/12 | 1/12 |
| ***IL21R*** | Hs.210546 | interleukin 21 receptor | 2/20 | 2/20 |
| ***IL2RB*** | Hs.474787 | interleukin 2 receptor, beta | 1/15 | 1/15 |
| ***IL7*** | Hs.591873 | interleukin 7 | 1/26 | 1/16 |
| ***ISLR2*** | Hs.254775 | immunoglobulin superfamily containing leucine-rich repeat 2 | 1/61 | 1/60 |
| ***ITGAL*** | Hs.174103 | integrin, alpha L (antigen CD11A (p180), lymphocyte function-associated antigen 1; alpha polypeptide) | 1/31 | 1/12 |
| ***ITGB2*** | Hs.375957 | integrin, beta 2 (complement component 3 receptor 3 and 4 subunit) | 3/62 | 2/60 |
| ***ITGB7*** | Hs.654470 | integrin, beta 7 | 4/20 | 4/16 |
| ***JAK3*** | Hs.515247 | Janus kinase 3 | 1/48 | 1/35 |
| ***JSRP1*** | Hs.712901 | junctional sarcoplasmic reticulum protein 1 | 4/69 | 0/48 |
| ***KCNA4*** | Hs.592002 | potassium voltage-gated channel, shaker-related subfamily, member 4 | 8/28 | 8/28 |
| ***KCNA7*** | Hs.306973 | potassium voltage-gated channel, shaker-related subfamily, member 7 | 2/25 | 2/14 |
| ***KCNG1*** | Hs.118695 | potassium voltage-gated channel, subfamily G, member 1 | 1/30 | 1/27 |
| ***KCNIP4*** | Hs.655705 | Kv channel interacting protein 4 | 5/58 | 5/57 |
| ***KCNJ15*** | Hs.411299 | potassium inwardly-rectifying channel, subfamily J, member 15 | 1/22 | 1/22 |
| ***KCNJ5*** | Hs.632109 | potassium inwardly-rectifying channel, subfamily J, member 5 | 1/43 | 0/41 |
| ***KCNN2*** | Hs.98280 | potassium intermediate/small conductance calcium-activated channel, subfamily N, member 2 | 4/29 | 4/29 |
| ***KCNN4*** | Hs.10082 | potassium intermediate/small conductance calcium-activated channel, subfamily N, member 4 | 3/16 | 3/15 |
| ***KIAA0513*** | Hs.301658 | KIAA0513 | 1/48 | 1/46 |
| ***KIAA1217*** | Hs.445885 | KIAA1217 | 2/71 | 2/70 |
| ***KIAA1598*** | Hs.501140 | KIAA1598 | 3/51 | 3/50 |
| ***KIF21B*** | Hs.169182 | kinesin family member 21B | 1/37 | 1/37 |
| ***KIF26B*** | Hs.368096 | kinesin family member 26B | 4/150 | 4/147 |
| ***KISS1*** | Hs.95008 | KiSS-1 metastasis-suppressor | 1/15 | 0/13 |
| ***KLHDC7B*** | Hs.137007 | kelch domain containing 7B | 1/23 | 1/20 |
| ***LAMA4*** | Hs.654572 | laminin, alpha 4 | 4/26 | 4/25 |
| ***LAMP3*** | Hs.518448 | lysosomal-associated membrane protein 3 | 1/18 | 1/15 |
| ***LAPTM5*** | Hs.371021 | lysosomal protein transmembrane 5 | 4/24 | 4/15 |
| ***LARGE*** | Hs.474667 | like-glycosyltransferase | 1/17 | 1/17 |
| ***LCP1*** | Hs.381099 | lymphocyte cytosolic protein 1 (L-plastin) | 1/29 | 1/29 |
| ***LDLRAD2*** | Hs.710255 | low density lipoprotein receptor class A domain containing 2 | 1/29 | 1/24 |
| ***LEF1*** | Hs.555947 | lymphoid enhancer-binding factor 1 | 1/39 | 1/39 |
| ***LGI4*** | Hs.65256 | leucine-rich repeat LGI family, member 4 | 1/56 | 1/42 |
| ***LILRA2*** | Hs.655593 | leukocyte immunoglobulin-like receptor, subfamily A (with TM domain), member 2 | 1/11 | 0/6 |
| ***LILRB1*** | Hs.667388 | leukocyte immunoglobulin-like receptor, subfamily B (with TM and ITIM domains), member 1 | 1/23 | 1/23 |
| ***LINC00426*** |  | long intergenic non-protein coding RNA 426 | 1/12 | 1/10 |
| ***LRRC25*** | Hs.332156 | leucine rich repeat containing 25 | 1/22 | 1/7 |
| ***LRRC4C*** | Hs.135736 | leucine rich repeat containing 4C | 4/45 | 4/45 |
| ***LSP1*** | Hs.56729 | lymphocyte-specific protein 1 | 8/61 | 8/56 |
| ***LST1*** | Hs.436066 | leukocyte specific transcript 1 | 5/76 | 5/66 |
| ***LTB*** | Hs.376208 | lymphotoxin beta (TNF superfamily, member 3) | 11/102 | 1/46 |
| ***MAFB*** | Hs.169487 | v-maf musculoaponeurotic fibrosarcoma oncogene homolog B (avian) | 3/35 | 1/27 |
| ***MAP4K1*** | Hs.95424 | mitogen-activated protein kinase kinase kinase kinase 1 | 1/27 | 1/26 |
| ***MDK*** | Hs.82045 | midkine (neurite growth-promoting factor 2) | 1/43 | 0/22 |
| ***MEX3D*** | Hs.436495 | mex-3 homolog D (C. elegans) | 1/32 | 1/24 |
| ***MFAP4*** | Hs.296049 | microfibrillar-associated protein 4 | 1/33 | 0/12 |
| ***MFSD4*** | Hs.567714 | major facilitator superfamily domain containing 4 | 2/32 | 2/31 |
| ***MICAL1*** | Hs.33476 | microtubule associated monoxygenase, calponin and LIM domain containing 1 | 1/28 | 1/20 |
| ***MIR155HG*** | Hs.697120 | MIR155 host gene (non-protein coding) | 2/14 | 2/12 |
| ***MLXIPL*** | Hs.647055 | MLX interacting protein-like | 1/34 | 1/31 |
| ***MMP25*** | Hs.654979 | matrix metallopeptidase 25 | 1/41 | 0/30 |
| ***MPEG1*** | Hs.730677 | macrophage expressed 1 | 2/10 | 2/9 |
| ***MSX1*** | Hs.424414 | msh homeobox 1 | 3/113 | 0/77 |
| ***MT1F*** | Hs.513626 | metallothionein 1F | 3/41 | 0/21 |
| ***MTMR11*** | Hs.425144 | myotubularin related protein 11 | 1/24 | 1/14 |
| ***MXRA5*** | Hs.369422 | matrix-remodelling associated 5 | 1/29 | 1/28 |
| ***MYO1F*** | Hs.465818 | myosin IF | 4/54 | 3/40 |
| ***MYOG*** | Hs.2830 | myogenin (myogenic factor 4) | 1/22 | 0/14 |
| ***MYRIP*** | Hs.594535 | myosin VIIA and Rab interacting protein | 1/23 | 1/23 |
| ***MZB1*** | Hs.409563 | marginal zone B and B1 cell-specific protein | 2/39 | 2/24 |
| ***NACC2*** | Hs.112895 | NACC family member 2, BEN and BTB (POZ) domain containing | 3/45 | 3/41 |
| ***NAV1*** | Hs.585374 | neuron navigator 1 | 4/89 | 4/77 |
| ***NCF4*** | Hs.474781 | neutrophil cytosolic factor 4, 40kDa | 1/13 | 1/13 |
| ***NCK2*** | Hs.529244 | NCK adaptor protein 2 | 1/56 | 1/55 |
| ***NCR3*** | Hs.509513 | natural cytotoxicity triggering receptor 3 | 1/63 | 0/10 |
| ***NFATC2*** | Hs.713650 | nuclear factor of activated T-cells, cytoplasmic, calcineurin-dependent 2 | 2/37 | 2/37 |
| ***NFE2L3*** | Hs.404741 | nuclear factor (erythroid-derived 2)-like 3 | 1/27 | 0/22 |
| ***NIPSNAP3B*** | Hs.429294 | nipsnap homolog 3B (C. elegans) | 1/9 | 1/8 |
| ***NKG7*** | Hs.10306 | natural killer cell group 7 sequence | 1/31 | 1/10 |
| ***NLRC3*** | Hs.728268 | NLR family, CARD domain containing 3 | 2/34 | 2/32 |
| ***NLRP3*** | Hs.159483 | NLR family, pyrin domain containing 3 | 1/37 | 1/24 |
| ***NOTCH2*** | Hs.487360 | notch 2 | 1/13 | 1/13 |
| ***NPAS3*** | Hs.657892 | neuronal PAS domain protein 3 | 1/61 | 1/61 |
| ***NRADDP*** |  | neurotrophin receptor associated death domain, pseudogene | 4/15 | 4/13 |
| ***NRK*** | Hs.209527 | Nik related kinase | 2/15 | 2/15 |
| ***NUP210*** | Hs.475525 | nucleoporin 210kDa | 1/26 | 1/26 |
| ***OSBPL6*** | Hs.318775 | oxysterol binding protein-like 6 | 1/31 | 0/30 |
| ***OTUD7A*** | Hs.355236 | OTU domain containing 7A | 1/30 | 1/27 |
| ***P2RY2*** | Hs.339 | purinergic receptor P2Y, G-protein coupled, 2 | 1/23 | 1/21 |
| ***PAPLN*** | Hs.509909 | papilin, proteoglycan-like sulfated glycoprotein | 2/29 | 2/27 |
| ***PAQR5*** | Hs.591096 | progestin and adipoQ receptor family member V | 1/46 | 1/29 |
| ***PARD6B*** | Hs.589848 | par-6 partitioning defective 6 homolog beta (C. elegans) | 1/17 | 1/17 |
| ***PARP10*** | Hs.348609 | poly (ADP-ribose) polymerase family, member 10 | 1/62 | 1/41 |
| ***PARP4*** | Hs.117825 | poly (ADP-ribose) polymerase family, member 4 | 2/21 | 2/21 |
| ***PARVG*** | Hs.658995 | parvin, gamma | 3/26 | 3/26 |
| ***PCDH20*** | Hs.391781 | protocadherin 20 | 2/20 | 1/20 |
| ***PCP4L1*** | Hs.433150 | Purkinje cell protein 4 like 1 | 1/12 | 1/12 |
| ***PCYOX1L*** | Hs.644397 | prenylcysteine oxidase 1 like | 1/27 | 0/17 |
| ***PDE4D*** | Hs.117545 | phosphodiesterase 4D, cAMP-specific | 4/89 | 4/89 |
| ***PDE4DIP*** | Hs.708687 | phosphodiesterase 4D interacting protein | 1/72 | 1/72 |
| ***PDE9A*** | Hs.473927 | phosphodiesterase 9A | 2/33 | 2/33 |
| ***PDIA2*** | Hs.66581 | protein disulfide isomerase family A, member 2 | 1/49 | 1/34 |
| ***PENK*** | Hs.339831 | proenkephalin | 7/38 | 7/37 |
| ***PHF21B*** | Hs.254097 | PHD finger protein 21B | 2/38 | 1/38 |
| ***PIP4K2A*** | Hs.57079 | phosphatidylinositol-5-phosphate 4-kinase, type II, alpha | 1/42 | 1/42 |
| ***PITX1*** | Hs.84136 | paired-like homeodomain 1 | 5/48 | 5/42 |
| ***PKLR*** | Hs.95990 | pyruvate kinase, liver and RBC | 1/38 | 1/34 |
| ***PLCB2*** | Hs.355888 | phospholipase C, beta 2 | 1/37 | 1/34 |
| ***PLD4*** | Hs.407101 | phospholipase D family, member 4 | 1/19 | 1/14 |
| ***PLXDC2*** | Hs.658134 | plexin domain containing 2 | 2/33 | 2/33 |
| ***POU2AF1*** | Hs.654525 | POU class 2 associating factor 1 | 2/25 | 2/25 |
| ***PPM1M*** | Hs.373560 | protein phosphatase, Mg2+/Mn2+ dependent, 1M | 1/30 | 1/27 |
| ***PPM1N*** | Hs.532872 | protein phosphatase, Mg2+/Mn2+ dependent, 1N (putative) | 1/42 | 1/30 |
| ***PPP1CC*** | Hs.79081 | protein phosphatase 1, catalytic subunit, gamma isozyme | 1/19 | 1/19 |
| ***PRAM1*** | Hs.465812 | PML-RARA regulated adaptor molecule 1 | 1/38 | 0/29 |
| ***PRDX6*** | Hs.120 | peroxiredoxin 6 | 1/16 | 1/15 |
| ***PRF1*** | Hs.2200 | perforin 1 (pore forming protein) | 6/18 | 6/18 |
| ***PRLHR*** | Hs.248119 | prolactin releasing hormone receptor | 6/18 | 6/17 |
| ***PRSS57*** | Hs.245146 | protease, serine, 57 | 1/34 | 1/19 |
| ***PSD4*** | Hs.516306 | pleckstrin and Sec7 domain containing 4 | 1/35 | 1/33 |
| ***PSTPIP1*** | Hs.129758 | proline-serine-threonine phosphatase interacting protein 1 | 3/24 | 3/23 |
| ***PSTPIP2*** | Hs.567384 | proline-serine-threonine phosphatase interacting protein 2 | 2/21 | 2/21 |
| ***PTDSS1*** | Hs.292579 | phosphatidylserine synthase 1 | 1/32 | 1/30 |
| ***PTK6*** | Hs.51133 | PTK6 protein tyrosine kinase 6 | 3/34 | 3/20 |
| ***PTPN22*** | Hs.535276 | protein tyrosine phosphatase, non-receptor type 22 (lymphoid) | 1/23 | 1/9 |
| ***PTPN6*** | Hs.63489 | protein tyrosine phosphatase, non-receptor type 6 | 9/75 | 9/44 |
| ***PTPN7*** | Hs.402773 | protein tyrosine phosphatase, non-receptor type 7 | 6/32 | 6/19 |
| ***PTPRC*** | Hs.654514 | protein tyrosine phosphatase, receptor type, C | 1/14 | 1/14 |
| ***PTPRCAP*** | Hs.155975 | protein tyrosine phosphatase, receptor type, C-associated protein | 11/57 | 10/38 |
| ***PTPRH*** | Hs.179770 | protein tyrosine phosphatase, receptor type, H | 1/35 | 0/12 |
| ***PTPRO*** | Hs.160871 | protein tyrosine phosphatase, receptor type, O | 1/24 | 1/24 |
| ***PTPRVP*** | Hs.523870 | protein tyrosine phosphatase, receptor type, V, pseudogene | 6/27 | 6/19 |
| ***PVT1*** | Hs.133107 | Pvt1 oncogene (non-protein coding) | 3/88 | 3/86 |
| ***PYDC1*** | Hs.58314 | PYD (pyrin domain) containing 1 | 1/31 | 0/23 |
| ***PYGO1*** | Hs.256587 | pygopus homolog 1 (Drosophila) | 1/18 | 1/18 |
| ***RAB30*** | Hs.40758 | RAB30, member RAS oncogene family | 1/24 | 1/23 |
| ***RAB37*** | Hs.351413 | RAB37, member RAS oncogene family | 2/53 | 2/41 |
| ***RAB8B*** | Hs.389733 | RAB8B, member RAS oncogene family | 1/26 | 1/15 |
| ***RANBP17*** | Hs.410810 | RAN binding protein 17 | 6/36 | 3/18 |
| ***RAP1GAP2*** | Hs.499659 | RAP1 GTPase activating protein 2 | 3/76 | 3/75 |
| ***RARRES3*** | Hs.17466 | retinoic acid receptor responder (tazarotene induced) 3 | 2/9 | 2/6 |
| ***RASAL3*** | Hs.136979 | RAS protein activator like 3 | 2/45 | 2/28 |
| ***RASEF*** | Hs.657750 | RAS and EF-hand domain containing | 1/7 | 1/7 |
| ***RASL10B*** | Hs.437035 | RAS-like, family 10, member B | 2/32 | 2/23 |
| ***RGS10*** | Hs.501200 | regulator of G-protein signaling 10 | 1/31 | 1/27 |
| ***RGS9BP*** | Hs.528491 | regulator of G protein signaling 9 binding protein | 1/22 | 1/20 |
| ***RHEB*** | Hs.647068 | Ras homolog enriched in brain | 2/33 | 0/30 |
| ***RHOBTB1*** | Hs.148670 | Rho-related BTB domain containing 1 | 3/38 | 3/38 |
| ***RHOD*** | Hs.15114 | ras homolog gene family, member D | 1/24 | 1/23 |
| ***RHOH*** | Hs.654594 | ras homolog gene family, member H | 2/11 | 2/11 |
| ***RLTPR*** | Hs.611432 | RGD motif, leucine rich repeats, tropomodulin domain and proline-rich containing | 1/66 | 1/26 |
| ***RMI2*** | Hs.347524 | RMI2, RecQ mediated genome instability 2, homolog (S. cerevisiae) | 1/91 | 1/89 |
| ***RNF213*** | Hs.195642 | ring finger protein 213 | 2/89 | 2/87 |
| ***RPH3A*** | Hs.21239 | rabphilin 3A homolog (mouse) | 2/42 | 2/35 |
| ***RPL27A*** | Hs.523463 | ribosomal protein L27a | 2/31 | 1/27 |
| ***RRAD*** | Hs.1027 | Ras-related associated with diabetes | 1/38 | 1/32 |
| ***RTN4RL1*** | Hs.22917 | reticulon 4 receptor-like 1 | 1/66 | 1/55 |
| ***RUNX2*** | Hs.535845 | runt-related transcription factor 2 | 2/89 | 2/88 |
| ***RUNX3*** | Hs.170019 | runt-related transcription factor 3 | 12/95 | 12/94 |
| ***RYR1*** | Hs.466664 | ryanodine receptor 1 (skeletal) | 1/78 | 0/72 |
| ***S1PR4*** | Hs.662006 | sphingosine-1-phosphate receptor 4 | 4/36 | 4/18 |
| ***SCG5*** | Hs.156540 | secretogranin V (7B2 protein) | 3/19 | 3/19 |
| ***SCML4*** | Hs.486109 | sex comb on midleg-like 4 (Drosophila) | 1/23 | 1/22 |
| ***SELPLG*** | Hs.591014 | selectin P ligand | 4/14 | 4/12 |
| ***SEMA4D*** | Hs.494406 | sema domain, immunoglobulin domain (Ig), transmembrane domain (TM) and short cytoplasmic domain, (semaphorin) 4D | 1/22 | 1/20 |
| ***SFI1*** | Hs.62209 | Sfi1 homolog, spindle assembly associated (yeast) | 1/31 | 1/27 |
| ***SFMBT2*** | Hs.407983 | Scm-like with four mbt domains 2 | 2/70 | 2/70 |
| ***SH2D1A*** | Hs.349094 | SH2 domain containing 1A | 1/7 | 1/7 |
| ***SH2D3A*** | Hs.439645 | SH2 domain containing 3A | 1/29 | 0/18 |
| ***SH3TC1*** | Hs.479116 | SH3 domain and tetratricopeptide repeats 1 | 3/48 | 3/37 |
| ***SHC4*** | Hs.642615 | SHC (Src homology 2 domain containing) family, member 4 | 1/45 | 1/44 |
| ***SIGLEC1*** | Hs.31869 | sialic acid binding Ig-like lectin 1, sialoadhesin | 1/21 | 1/11 |
| ***SLA*** | Hs.75367 | Src-like-adaptor | 3/26 | 3/23 |
| ***SLA2*** | Hs.713578 | Src-like-adaptor 2 | 1/23 | 1/14 |
| ***SLAMF1*** | Hs.523660 | signaling lymphocytic activation molecule family member 1 | 3/11 | 3/11 |
| ***SLAMF8*** | Hs.438683 | SLAM family member 8 | 3/13 | 3/13 |
| ***SLC25A34*** | Hs.631867 | solute carrier family 25, member 34 | 1/48 | 0/28 |
| ***SLC4A8*** | Hs.370636 | solute carrier family 4, sodium bicarbonate cotransporter, member 8 | 2/41 | 2/41 |
| ***SLC7A7*** | Hs.513147 | solute carrier family 7 (amino acid transporter light chain, y+L system), member 7 | 5/66 | 5/51 |
| ***SLFN12L*** | Hs.447559 | schlafen family member 12-like | 5/37 | 4/30 |
| ***SLIT2*** | Hs.29802 | slit homolog 2 (Drosophila) | 3/30 | 3/30 |
| ***SLITRK4*** | Hs.272284 | SLIT and NTRK-like family, member 4 | 3/24 | 3/24 |
| ***SMOC2*** | Hs.487200 | SPARC related modular calcium binding 2 | 5/169 | 5/168 |
| ***SNX20*** | Hs.715778 | sorting nexin 20 | 1/19 | 1/16 |
| ***SOCS1*** | Hs.50640 | suppressor of cytokine signaling 1 | 1/34 | 0/27 |
| ***SORCS1*** | Hs.591915 | sortilin-related VPS10 domain containing receptor 1 | 2/24 | 2/23 |
| ***SP140*** | Hs.632549 | SP140 nuclear body protein | 2/20 | 2/20 |
| ***SP140L*** | Hs.589661 | SP140 nuclear body protein-like | 1/15 | 1/8 |
| ***SPATA24*** | Hs.373612 | spermatogenesis associated 24 | 2/39 | 0/16 |
| ***SPC24*** | Hs.381225 | SPC24, NDC80 kinetochore complex component, homolog (S. cerevisiae) | 1/16 | 1/16 |
| ***SRGAP2*** | Hs.497575 | SLIT-ROBO Rho GTPase activating protein 2 | 1/21 | 0/13 |
| ***SRRM3*** | Hs.511025 | serine/arginine repetitive matrix 3 | 2/57 | 2/56 |
| ***STAG3*** | Hs.592283 | stromal antigen 3 | 7/43 | 7/43 |
| ***STAT1*** | Hs.642990 | signal transducer and activator of transcription 1, 91kDa | 3/26 | 3/25 |
| ***SUSD4*** | Hs.497841 | sushi domain containing 4 | 1/21 | 1/21 |
| ***SYNGAP1*** | Hs.586264 | synaptic Ras GTPase activating protein 1 | 2/273 | 2/218 |
| ***SYPL2*** | Hs.528366 | synaptophysin-like 2 | 1/24 | 1/14 |
| ***SYT7*** | Hs.502730 | synaptotagmin VII | 3/72 | 3/60 |
| ***SYTL1*** | Hs.469175 | synaptotagmin-like 1 | 5/43 | 3/25 |
| ***TACSTD2*** | Hs.23582 | tumor-associated calcium signal transducer 2 | 2/20 | 2/18 |
| ***TBC1D10C*** | Hs.534648 | TBC1 domain family, member 10C | 4/52 | 4/38 |
| ***TCEANC*** | Hs.222855 | transcription elongation factor A (SII) N-terminal and central domain containing | 1/23 | 0/17 |
| ***TESC*** | Hs.525709 | tescalcin | 1/60 | 1/44 |
| ***TFCP2L1*** | Hs.156471 | transcription factor CP2-like 1 | 1/27 | 1/24 |
| ***TGFBR2*** | Hs.82028 | transforming growth factor, beta receptor II (70/80kDa) | 1/37 | 1/36 |
| ***THY1*** | Hs.644697 | Thy-1 cell surface antigen | 1/21 | 1/21 |
| ***TIFAB*** | Hs.552091 | TRAF-interacting protein with forkhead-associated domain, family member B | 1/18 | 1/17 |
| ***TLCD1*** | Hs.499952 | TLC domain containing 1 | 3/64 | 0/24 |
| ***TLR6*** | Hs.575090 | toll-like receptor 6 | 2/11 | 2/11 |
| ***TMC8*** | Hs.592102 | transmembrane channel-like 8 | 5/52 | 5/45 |
| ***TMEM132E*** | Hs.310482 | transmembrane protein 132E | 3/51 | 3/49 |
| ***TMEM171*** | Hs.162246 | transmembrane protein 171 | 2/16 | 2/15 |
| ***TMEM189*** | Hs.420529 | transmembrane protein 189 | 1/36 | 1/36 |
| ***TMPRSS3*** | Hs.208600 | transmembrane protease, serine 3 | 1/28 | 1/19 |
| ***TNFAIP8L2*** | Hs.432360 | tumor necrosis factor, alpha-induced protein 8-like 2 | 1/39 | 0/17 |
| ***TNFRSF19*** | Hs.730848 | tumor necrosis factor receptor superfamily, member 19 | 1/40 | 0/39 |
| ***TNFSF13B*** | Hs.525157 | tumor necrosis factor (ligand) superfamily, member 13b | 2/15 | 2/15 |
| ***TNFSF14*** | Hs.129708 | tumor necrosis factor (ligand) superfamily, member 14 | 1/13 | 0/7 |
| ***TNIP3*** | Hs.208206 | TNFAIP3 interacting protein 3 | 2/14 | 2/14 |
| ***TNNI2*** | Hs.523403 | troponin I type 2 (skeletal, fast) | 4/54 | 2/49 |
| ***TP53INP1*** | Hs.492261 | tumor protein p53 inducible nuclear protein 1 | 2/17 | 2/17 |
| ***TPD52*** | Hs.309921 | tumor protein D52 | 1/49 | 1/48 |
| ***TRAF1*** | Hs.531251 | TNF receptor-associated factor 1 | 3/17 | 2/13 |
| ***TRAF3IP3*** | Hs.147434 | TRAF3 interacting protein 3 | 6/36 | 6/18 |
| ***TRAF5*** | Hs.523930 | TNF receptor-associated factor 5 | 3/36 | 3/23 |
| ***TRERF1*** | Hs.485392 | transcriptional regulating factor 1 | 4/66 | 4/55 |
| ***TRIM14*** | Hs.575631 | tripartite motif containing 14 | 2/15 | 2/14 |
| ***TRIM4*** | Hs.50749 | tripartite motif containing 4 | 1/25 | 1/24 |
| ***TRIM66*** | Hs.130836 | tripartite motif containing 66 | 1/15 | 1/14 |
| ***TRIP13*** | Hs.436187 | thyroid hormone receptor interactor 13 | 2/49 | 1/42 |
| ***TRPC4*** | Hs.262960 | transient receptor potential cation channel, subfamily C, member 4 | 1/24 | 1/24 |
| ***TRPM2*** | Hs.369759 | transient receptor potential cation channel, subfamily M, member 2 | 4/43 | 4/42 |
| ***TSPAN5*** | Hs.118118 | tetraspanin 5 | 1/46 | 1/42 |
| ***TTC24*** | Hs.447851 | tetratricopeptide repeat domain 24 | 2/34 | 2/23 |
| ***TTYH1*** | Hs.268728 | tweety homolog 1 (Drosophila) | 1/18 | 1/17 |
| ***TUSC1*** | Hs.26268 | tumor suppressor candidate 1 | 1/7 | 1/7 |
| ***TYROBP*** | Hs.515369 | TYRO protein tyrosine kinase binding protein | 1/26 | 0/9 |
| ***UBASH3A*** | Hs.473912 | ubiquitin associated and SH3 domain containing A | 1/16 | 1/16 |
| ***UBD*** | Hs.728313 | ubiquitin D | 10/65 | 0/25 |
| ***UMOD*** | Hs.654425 | uromodulin | 1/18 | 1/14 |
| ***VAC14*** | Hs.445061 | Vac14 homolog (S. cerevisiae) | 1/64 | 1/45 |
| ***WDFY4*** | Hs.287379 | WDFY family member 4 | 5/43 | 5/43 |
| ***WIPF1*** | Hs.128067 | WAS/WASL interacting protein family, member 1 | 2/41 | 2/41 |
| ***WNT3A*** | Hs.336930 | wingless-type MMTV integration site family, member 3A | 1/39 | 1/39 |
| ***WSCD1*** | Hs.370166 | WSC domain containing 1 | 1/55 | 1/55 |
| ***WWC1*** | Hs.484047 | WW and C2 domain containing 1 | 1/35 | 1/35 |
| ***XAF1*** | Hs.441975 | XIAP associated factor 1 | 5/28 | 0/15 |
| ***XRCC3*** | Hs.592325 | X-ray repair complementing defective repair in Chinese hamster cells 3 | 1/55 | 1/47 |
| ***ZACN*** | Hs.714919 | zinc activated ligand-gated ion channel | 2/43 | 2/38 |
| ***ZFHX4*** | Hs.458973 | zinc finger homeobox 4 | 2/68 | 2/68 |
| ***ZMYND15*** | Hs.47223 | zinc finger, MYND-type containing 15 | 1/37 | 1/37 |
| ***ZNF214*** | Hs.445849 | zinc finger protein 214 | 1/20 | 1/19 |
| ***ZNF217*** | Hs.155040 | zinc finger protein 217 | 7/23 | 7/23 |
| ***ZNF831*** | Hs.473204 | zinc finger protein 831 | 1/24 | 1/24 |
| ***ZNRD1-AS1*** | Hs.653168 | ZNRD1 antisense RNA 1 (non-protein coding) | 12/303 | 11/303 |

1. Number of differentially methylated CpGs located in the gene unit compared to the number of CpGs tested and located in the gene unit.
2. Number of differentially methylated CpGs located in the 10Kb proximal promoter region compared to the number of CpGs tested and located in the 10Kb proximal promoter region.
