# Supplementary material for: Whole-Genome Cardiac DNA Methylation Fingerprint and Gene Expression Analysis Provide New Insights in the Pathogenesis of Chronic Chagas Disease Cardiomyopathy
Source: Clin Infect Dis. 2017 May 30;65(7):1103–11. doi: 10.1093/cid/cix506 (PMC5849099; doi:10.1093/cid/cix506)
Supplement: Supplementary_table_4_20170516 [file cix506_suppl_supplementary_table_4_20170516.docx]

**Supplementary table 4:** Go ontology analysis of the 399 genes differentially expressed and differentially methylated in heart tissue samples.

| **GO ontology**  **(cellular component)** | **Percentage of**  **concerned gene** | **Fold Enrichment** | **P value** |
| --- | --- | --- | --- |
| plasma membrane | 3.4 (172/5015) | 1.81 | 2.79E-14 |
| cell periphery | 3.4 (173/5118) | 1.78 | 9.10E-14 |
| plasma membrane part | 4.1 (107/2579) | 2.19 | 3.97E-12 |
| membrane | 2.5 (238/9350) | 1.34 | 9.83E-07 |
| plasma membrane protein complex | 6.4 (33/513) | 3.39 | 2.46E-06 |
| membrane part | 2.7 (190/7057) | 1.42 | 5.56E-06 |
| intrinsic component of plasma membrane | 4.0 (66/1649) | 2.11 | 1.02E-05 |
| side of membrane | 6.6 (29/440) | 3.47 | 1.44E-05 |
| cellular_component | 2.1 (377/18125) | 1.10 | 6.88E-05 |
| cell surface | 5.0 (38/764) | 2.62 | 1.26E-04 |
| T cell receptor complex | 36.8 (7/19) | 19.41 | 1.37E-04 |
| integral component of plasma membrane | 3.8 (60/1587) | 1.99 | 3.78E-04 |
| intrinsic component of membrane | 2.7 (160/5992) | 1.41 | 5.34E-04 |
| mitochondrion | 0.5 (9/1681) | 0.28 | 1.11E-03 |
| MHC protein complex | 25.0 (7/28) | 13.17 | 1.79E-03 |
| external side of plasma membrane | 7.4 (18/243) | 3.90 | 1.83E-03 |
| phagocytic vesicle | 12.4 (11/89) | 6.51 | 1.94E-03 |
| integral component of membrane | 2.6 (155/5883) | 1.39 | 2.22E-03 |
| MHC class II protein complex | 30.0 (6/20) | 15.81 | 3.64E-03 |
| alpha-beta T cell receptor complex | 80.0 (4/5) | 42.15 | 3.86E-03 |
| mitochondrial part | 0.3 (3/981) | < 0.2 | 9.47E-03 |
| plasma membrane receptor complex | 8.1 (14/172) | 4.29 | 9.50E-03 |
| nucleoplasm | 1.0 (29/2924) | 0.52 | 3.10E-02 |
| binding | 2.3 (325/14268) | 1.20 | 1.77E-06 |
| protein binding | 2.4 (258/10751) | 1.26 | 8.31E-05 |
| receptor activity | 3.9 (64/1661) | 2.03 | 1.49E-04 |
| molecular transducer activity | 3.9 (64/1661) | 2.03 | 1.49E-04 |
| receptor binding | 3.9 (58/1502) | 2.03 | 6.40E-04 |
| RNA binding | 0.6 (10/1617) | 0.33 | 2.02E-02 |
| molecular_function | 2.1 (355/17018) | 1.10 | 2.20E-02 |
| transferase activity | 0.9 (21/2417) | 0.46 | 3.51E-02 |
