# Supplementary material for: Whole-Genome Cardiac DNA Methylation Fingerprint and Gene Expression Analysis Provide New Insights in the Pathogenesis of Chronic Chagas Disease Cardiomyopathy
Source: Clin Infect Dis. 2017 May 30;65(7):1103–11. doi: 10.1093/cid/cix506 (PMC5849099; doi:10.1093/cid/cix506)
Supplement: Supplementary_table_5_20170516 [file cix506_suppl_supplementary_table_5_20170516.docx]

**Supplementary table 5:** Biological process of the 399 genes differentially expressed and differentially methylated in heart tissue biopsies.

| **GO ontology**  **(biological process)** | **Percentage of**  **concerned gene** | **Fold Enrichment** | **P value** |
| --- | --- | --- | --- |
| immune system process | 5.3 (107/2004) | 2.81 | 3.28E-19 |
| positive regulation of cell-cell adhesion | 13.8 (34/246) | 7.28 | 5.62E-15 |
| regulation of immune system process | 5.6 (78/1384) | 2.97 | 5.11E-14 |
| regulation of lymphocyte activation | 10.1 (41/404) | 5.35 | 6.14E-14 |
| positive regulation of lymphocyte activation | 12.2 (35/288) | 6.40 | 8.43E-14 |
| leukocyte activation | 9.8 (41/418) | 5.17 | 1.96E-13 |
| positive regulation of T cell activation | 14.4 (30/209) | 7.56 | 2.52E-13 |
| positive regulation of cell adhesion | 10.2 (39/384) | 5.35 | 3.97E-13 |
| positive regulation of leukocyte cell-cell adhesion | 13.8 (30/217) | 7.28 | 6.79E-13 |
| regulation of leukocyte activation | 9.2 (42/459) | 4.82 | 8.59E-13 |
| positive regulation of leukocyte activation | 11.2 (35/312) | 5.91 | 9.24E-13 |
| regulation of cell activation | 8.7 (43/492) | 4.61 | 1.80E-12 |
| positive regulation of cell activation | 10.9 (35/320) | 5.76 | 1.96E-12 |
| regulation of response to stimulus | 3.8 (136/3614) | 1.98 | 2.34E-12 |
| lymphocyte activation | 10.4 (36/346) | 5.48 | 3.25E-12 |
| regulation of cell-cell adhesion | 9.7 (37/381) | 5.12 | 1.04E-11 |
| regulation of leukocyte proliferation | 13.5 (28/208) | 7.09 | 1.43E-11 |
| single organism cell adhesion | 8.7 (40/461) | 4.57 | 2.69E-11 |
| single organismal cell-cell adhesion | 8.9 (39/439) | 4.68 | 2.90E-11 |
| regulation of lymphocyte proliferation | 13.7 (27/197) | 7.22 | 3.01E-11 |
| regulation of mononuclear cell proliferation | 13.6 (27/199) | 7.15 | 3.82E-11 |
| regulation of T cell activation | 10.9 (32/293) | 5.75 | 4.08E-11 |
| leukocyte cell-cell adhesion | 11.7 (30/256) | 6.18 | 4.97E-11 |
| immune response | 5.7 (63/1102) | 3.01 | 6.89E-11 |
| cell activation | 7.7 (44/573) | 4.05 | 7.24E-11 |
| positive regulation of leukocyte proliferation | 16.2 (23/142) | 8.53 | 1.14E-10 |
| regulation of leukocyte cell-cell adhesion | 10.5 (32/306) | 5.51 | 1.31E-10 |
| regulation of immune response | 6.3 (54/853) | 3.34 | 1.36E-10 |
| regulation of cell adhesion | 7.2 (46/639) | 3.79 | 1.69E-10 |
| cell-cell adhesion | 7.3 (45/617) | 3.84 | 2.12E-10 |
| response to stimulus | 2.9 (217/7529) | 1.52 | 2.38E-10 |
| positive regulation of lymphocyte proliferation | 16.4 (22/134) | 8.65 | 3.30E-10 |
| positive regulation of mononuclear cell proliferation | 16.2 (22/136) | 8.52 | 4.41E-10 |
| leukocyte aggregation | 11.9 (27/226) | 6.30 | 7.46E-10 |
| positive regulation of immune system process | 6.1 (53/869) | 3.21 | 1.03E-09 |
| cell adhesion | 5.6 (58/1037) | 2.95 | 2.27E-09 |
| biological adhesion | 5.6 (58/1042) | 2.93 | 2.76E-09 |
| defense response | 5.1 (63/1228) | 2.70 | 7.88E-09 |
| biological regulation | 2.5 (285/11384) | 1.32 | 8.23E-09 |
| T cell aggregation | 11.5 (25/218) | 6.04 | 1.50E-08 |
| T cell activation | 11.5 (25/218) | 6.04 | 1.50E-08 |
| regulation of biological process | 2.5 (273/10767) | 1.34 | 1.74E-08 |
| lymphocyte aggregation | 11.4 (25/220) | 5.99 | 1.82E-08 |
| single organism signaling | 3.1 (158/5074) | 1.64 | 4.67E-08 |
| signaling | 3.1 (158/5077) | 1.64 | 4.90E-08 |
| positive regulation of response to stimulus | 4.2 (80/1899) | 2.22 | 9.86E-08 |
| positive regulation of T cell proliferation | 17.2 (17/99) | 9.05 | 1.33E-07 |
| signal transduction | 3.1 (148/4744) | 1.64 | 3.19E-07 |
| cell communication | 3.0 (157/5164) | 1.60 | 3.98E-07 |
| regulation of cellular process | 2.5 (259/10292) | 1.33 | 7.31E-07 |
| regulation of T cell proliferation | 12.8 (19/148) | 6.76 | 1.12E-06 |
| regulation of multicellular organismal process | 3.7 (95/2589) | 1.93 | 1.50E-06 |
| cell surface receptor signaling pathway | 3.9 (83/2145) | 2.04 | 2.46E-06 |
| cellular response to stimulus | 2.8 (173/6098) | 1.49 | 6.11E-06 |
| positive regulation of immune response | 6.2 (35/566) | 3.26 | 1.37E-05 |
| regulation of signaling | 3.4 (103/3037) | 1.79 | 1.59E-05 |
| regulation of signal transduction | 3.5 (94/2690) | 1.84 | 2.38E-05 |
| regulation of cell communication | 3.4 (101/2987) | 1.78 | 2.84E-05 |
| cell migration | 5.4 (41/765) | 2.82 | 3.15E-05 |
| single-organism cellular process | 2.4 (270/11253) | 1.26 | 4.81E-05 |
| regulation of cytokine production | 6.0 (34/566) | 3.17 | 4.82E-05 |
| regulation of intracellular signal transduction | 4.0 (67/1677) | 2.11 | 5.31E-05 |
| regulation of cell proliferation | 4.1 (62/1515) | 2.16 | 9.00E-05 |
| regulation of localization | 3.5 (85/2418) | 1.85 | 1.31E-04 |
| positive regulation of cytokine production | 7.0 (26/373) | 3.67 | 1.82E-04 |
| localization of cell | 4.9 (41/842) | 2.57 | 4.32E-04 |
| cell motility | 4.9 (41/842) | 2.57 | 4.32E-04 |
| biological_process | 2.1 (359/16836) | 1.12 | 4.52E-04 |
| single-organism process | 2.3 (287/12451) | 1.21 | 6.50E-04 |
| positive regulation of biological process | 2.8 (143/5059) | 1.49 | 7.35E-04 |
| locomotion | 4.3 (46/1059) | 2.29 | 1.70E-03 |
| regulation of cytokine secretion | 10.1 (15/148) | 5.34 | 2.01E-03 |
| leukocyte differentiation | 7.0 (21/298) | 3.71 | 3.50E-03 |
| hematopoietic or lymphoid organ development | 5.3 (30/565) | 2.80 | 5.13E-03 |
| leukocyte migration | 7.3 (19/259) | 3.87 | 6.94E-03 |
| positive regulation of cell proliferation | 4.6 (38/835) | 2.40 | 7.01E-03 |
| hemopoiesis | 5.4 (28/514) | 2.87 | 7.50E-03 |
| activation of immune response | 5.8 (25/428) | 3.08 | 8.61E-03 |
| response to stress | 3 (99/3276) | 1.59 | 9.87E-03 |
| regulation of locomotion | 4.7 (35/747) | 2.47 | 1.02E-02 |
| regulation of transport | 3.5 (63/1784) | 1.86 | 1.18E-02 |
| positive regulation of cellular process | 2.8 (128/4604) | 1.46 | 1.33E-02 |
| immunological synapse formation | 50.0 (5/10) | 26.35 | 1.38E-02 |
| positive regulation of multicellular organismal process | 3.8 (53/1407) | 1.98 | 1.46E-02 |
| immune system development | 5.0 (30/601) | 2.63 | 1.76E-02 |
| positive regulation of secretion by cell | 6.3 (21/332) | 3.33 | 1.89E-02 |
| positive regulation of secretion | 6.1 (22/362) | 3.20 | 2.05E-02 |
| regulation of interferon-gamma production | 11.7 (11/94) | 6.17 | 2.10E-02 |
| regulation of cellular component movement | 4.5 (35/776) | 2.38 | 2.35E-02 |
| regulation of secretion | 4.7 (32/676) | 2.49 | 2.40E-02 |
| regulation of GTPase activity | 4.7 (32/680) | 2.48 | 2.71E-02 |
| movement of cell or subcellular component | 3.8 (49/1287) | 2.01 | 2.79E-02 |
| innate immune response | 4.9 (30/616) | 2.57 | 2.85E-02 |
| positive regulation of cytokine secretion | 11.2 (11/98) | 5.91 | 3.10E-02 |
| cytokine-mediated signaling pathway | 5.4 (25/461) | 2.86 | 3.13E-02 |
| regulation of leukocyte differentiation | 7.2 (17/236) | 3.80 | 3.31E-02 |
| regulation of secretion by cell | 4.8 (30/621) | 2.55 | 3.33E-02 |
| positive regulation of protein secretion | 7.6 (16/211) | 4.00 | 3.36E-02 |
| lymphocyte differentiation | 7.5 (16/213) | 3.96 | 3.78E-02 |
| positive regulation of interferon-gamma production | 13.8 (9/65) | 7.30 | 4.55E-02 |
