# Supplementary material for: Whole-Genome Cardiac DNA Methylation Fingerprint and Gene Expression Analysis Provide New Insights in the Pathogenesis of Chronic Chagas Disease Cardiomyopathy
Source: Clin Infect Dis. 2017 May 30;65(7):1103–11. doi: 10.1093/cid/cix506 (PMC5849099; doi:10.1093/cid/cix506)
Supplement: Supplementary_table_6_20170516 [file cix506_suppl_supplementary_table_6_20170516.docx]

**Supplementary table 6:** Linear regression analysis between expression data and methylation data (eQTL=f(mQTL)).

| **GENE** | **P value** |
| --- | --- |
| ***ABCC3*** | 3,53E-05 |
| ***ABLIM2*** | 7,09E-04 |
| ***ACAP1*** | 1,39E-03 |
| ***ADAM8*** | 6,78E-06 |
| ***ADARB2*** | 6,27E-03 |
| ***ADCY7*** | 1,22E-03 |
| ***ADORA3*** | 5,33E-05 |
| ***ADRB1*** | 6,35E-04 |
| ***AGAP3*** | 8,79E-04 |
| ***AIM1*** | 1,55E-06 |
| ***AIM1L*** | 4,09E-01 |
| ***AIM2*** | 8,43E-07 |
| ***ALOX15B*** | 3,99E-03 |
| ***AMICA1*** | 8,77E-07 |
| ***ANKRD36BP2*** | 6,65E-04 |
| ***ANKRD44*** | 3,19E-05 |
| ***APBA2*** | 8,04E-06 |
| ***APBB1IP*** | 2,73E-07 |
| ***APCDD1L*** | 1,40E-02 |
| ***APOBR*** | 4,71E-03 |
| ***AQP10*** | 8,44E-03 |
| ***ARAP2*** | 1,30E-04 |
| ***ARHGAP30*** | 2,10E-04 |
| ***ARHGAP4*** | 1,91E-04 |
| ***ARHGAP9*** | 2,11E-04 |
| ***ARHGEF16*** | 3,20E-03 |
| ***ASCL2*** | 7,84E-05 |
| ***ATP2A3*** | 4,89E-05 |
| ***ATP8A2*** | 5,47E-02 |
| ***ATP8B2*** | 1,52E-05 |
| ***ATXN7L1*** | 4,52E-03 |
| ***BATF*** | 2,11E-05 |
| ***BCAR1*** | 7,54E-03 |
| ***BHLHE22*** | 2,08E-06 |
| ***BMP7*** | 3,95E-04 |
| ***C10orf105*** | 2,06E-04 |
| ***C14orf182*** | 2,98E-06 |
| ***C16orf54*** | 4,89E-05 |
| ***C1QTNF1*** | 3,49E-03 |
| ***C1QTNF2*** | 4,80E-04 |
| ***C1QTNF6*** | 2,46E-03 |
| ***C1QTNF7*** | 1,15E-02 |
| ***C1S*** | 4,57E-04 |
| ***C2*** | 8,87E-05 |
| ***C5orf20*** | 1,61E-06 |
| ***C5orf56*** | 1,15E-03 |
| ***C7orf63*** | 5,65E-03 |
| ***C9orf3*** | 2,37E-05 |
| ***CACNA1B*** | 5,37E-04 |
| ***CACNA2D3*** | 1,35E-03 |
| ***CCDC85C*** | 1,03E-01 |
| ***CCDC88B*** | 1,49E-04 |
| ***CCDC88C*** | 1,38E-01 |
| ***CCL19*** | 8,91E-04 |
| ***CCR7*** | 1,94E-05 |
| ***CD160*** | 4,48E-04 |
| ***CD247*** | 6,60E-06 |
| ***CD300LF*** | 4,21E-05 |
| ***CD37*** | 2,35E-05 |
| ***CD3D*** | 1,86E-05 |
| ***CD3G*** | 1,88E-06 |
| ***CD48*** | 6,89E-07 |
| ***CD5*** | 3,53E-06 |
| ***CD52*** | 2,70E-04 |
| ***CD6*** | 3,19E-07 |
| ***CD74*** | 1,99E-04 |
| ***CD8A*** | 2,48E-07 |
| ***CD96*** | 2,46E-06 |
| ***CDH9*** | 1,43E-04 |
| ***CDX1*** | 1,82E-02 |
| ***CEBPE*** | 9,16E-05 |
| ***CGNL1*** | 8,05E-04 |
| ***CHDH*** | 5,25E-04 |
| ***CHST15*** | 8,62E-03 |
| ***CLDN14*** | 2,29E-04 |
| ***CLNK*** | 7,73E-07 |
| ***CNN1*** | 2,62E-03 |
| ***COL16A1*** | 8,77E-05 |
| ***COL5A1*** | 1,68E-02 |
| ***CORO1A*** | 2,42E-06 |
| ***CORO2A*** | 3,62E-05 |
| ***COTL1*** | 2,30E-04 |
| ***CPNE7*** | 1,23E-05 |
| ***CPXM2*** | 1,38E-04 |
| ***CXCL16*** | 6,61E-04 |
| ***CXCR6*** | 3,94E-07 |
| ***CYTH4*** | 5,66E-03 |
| ***DAPP1*** | 5,93E-06 |
| ***DCHS2*** | 3,00E-03 |
| ***DDX31*** | 1,43E-02 |
| ***DEF6*** | 4,08E-07 |
| ***DENND1C*** | 2,75E-08 |
| ***DENND2D*** | 5,26E-07 |
| ***DLEC1*** | 2,83E-01 |
| ***DLEU1*** | 1,71E-04 |
| ***DNAJB5*** | 1,79E-03 |
| ***DNALI1*** | 2,75E-03 |
| ***DNASE1L3*** | 2,61E-03 |
| ***DOK2*** | 8,62E-05 |
| ***DOK3*** | 3,66E-04 |
| ***DQX1*** | 5,36E-05 |
| ***DRD4*** | 6,16E-03 |
| ***DUSP13*** | 1,04E-03 |
| ***EBI3*** | 1,28E-03 |
| ***ERI2*** | 3,95E-02 |
| ***F2R*** | 1,90E-03 |
| ***FAAH*** | 6,08E-03 |
| ***FAM65B*** | 3,70E-05 |
| ***FAM78B*** | 4,94E-03 |
| ***FAM83B*** | 3,44E-03 |
| ***FANCA*** | 2,09E-05 |
| ***FASLG*** | 4,61E-06 |
| ***FBLIM1*** | 1,11E-05 |
| ***FCHO1*** | 3,85E-04 |
| ***FCN1*** | 4,89E-06 |
| ***FLT3LG*** | 4,63E-03 |
| ***FMNL1*** | 9,69E-05 |
| ***FNDC9*** | 3,46E-02 |
| ***FOLR2*** | 1,93E-02 |
| ***FOXP2*** | 5,24E-06 |
| ***FRZB*** | 4,58E-05 |
| ***FURIN*** | 1,82E-04 |
| ***GALNT6*** | 1,56E-03 |
| ***GATA4*** | 1,17E-01 |
| ***GFI1*** | 1,54E-09 |
| ***GFRA2*** | 8,39E-05 |
| ***GGN*** | 3,46E-02 |
| ***GHRL*** | 1,84E-05 |
| ***GLIS1*** | 2,01E-04 |
| ***GLIS2*** | 3,38E-02 |
| ***GNMT*** | 3,58E-03 |
| ***GPC6*** | 1,33E-05 |
| ***GPR160*** | 1,73E-03 |
| ***GPX3*** | 1,67E-03 |
| ***GRIK3*** | 1,40E-05 |
| ***GSDMB*** | 1,11E-03 |
| ***GTF2IRD1*** | 4,02E-04 |
| ***HAAO*** | 1,51E-03 |
| ***HCP5*** | 3,62E-04 |
| ***HCST*** | 3,30E-06 |
| ***HLA-DMA*** | 5,87E-05 |
| ***HLA-DMB*** | 6,85E-04 |
| ***HLA-DOB*** | 2,73E-03 |
| ***HLA-DPA1*** | 7,06E-03 |
| ***HLA-DPB1*** | 7,05E-04 |
| ***HLA-F*** | 1,90E-04 |
| ***HLA-J*** | 3,03E-03 |
| ***HMHA1*** | 6,89E-05 |
| ***HOOK1*** | 4,66E-04 |
| ***ICAM4*** | 1,35E-04 |
| ***IGSF21*** | 9,51E-03 |
| ***IGSF22*** | 1,09E-02 |
| ***IKZF3*** | 8,80E-07 |
| ***IL12RB1*** | 1,37E-04 |
| ***IL16*** | 1,11E-04 |
| ***IL17RB*** | 2,10E-02 |
| ***IL21R*** | 2,09E-06 |
| ***IL2RB*** | 7,22E-07 |
| ***IL7*** | 2,44E-06 |
| ***ISLR2*** | 3,35E-02 |
| ***ITGAL*** | 6,90E-07 |
| ***ITGB2*** | 1,58E-04 |
| ***ITGB7*** | 2,74E-08 |
| ***JAK3*** | 8,64E-04 |
| ***JSRP1*** | 8,43E-04 |
| ***KCNA4*** | 2,44E-03 |
| ***KCNA7*** | 2,74E-03 |
| ***KCNG1*** | 4,23E-03 |
| ***KCNIP4*** | 1,64E-04 |
| ***KCNJ15*** | 2,99E-04 |
| ***KCNJ5*** | 2,66E-03 |
| ***KCNN2*** | 7,52E-05 |
| ***KCNN4*** | 1,29E-01 |
| ***KIAA0513*** | 1,23E-03 |
| ***KIAA1217*** | 8,44E-02 |
| ***KIAA1598*** | 1,81E-03 |
| ***KIF21B*** | 8,87E-04 |
| ***KIF26B*** | 2,90E-02 |
| ***KISS1*** | 2,11E-02 |
| ***KLHDC7B*** | 1,43E-04 |
| ***LAMA4*** | 1,88E-05 |
| ***LAMP3*** | 5,27E-05 |
| ***LAPTM5*** | 6,14E-04 |
| ***LARGE*** | 1,80E-05 |
| ***LCP1*** | 5,54E-05 |
| ***LDLRAD2*** | 1,69E-04 |
| ***LEF1*** | 3,56E-04 |
| ***LGI4*** | 2,88E-03 |
| ***LILRA2*** | 1,47E-03 |
| ***LILRB1*** | 5,39E-05 |
| ***LINC00426*** | 9,44E-09 |
| ***LRRC25*** | 7,36E-04 |
| ***LRRC4C*** | 9,63E-04 |
| ***LSP1*** | 1,38E-03 |
| ***LST1*** | 2,41E-05 |
| ***LTB*** | 2,92E-05 |
| ***MAFB*** | 3,77E-01 |
| ***MAP4K1*** | 1,42E-06 |
| ***MDK*** | 1,66E-02 |
| ***MEX3D*** | 5,17E-05 |
| ***MFAP4*** | 7,32E-04 |
| ***MFSD4*** | 9,78E-03 |
| ***MICAL1*** | 1,03E-04 |
| ***MIR155HG*** | 5,77E-04 |
| ***MLXIPL*** | 3,51E-03 |
| ***MMP25*** | 5,83E-04 |
| ***MPEG1*** | 2,40E-05 |
| ***MSX1*** | 1,11E-03 |
| ***MT1F*** | 1,79E-03 |
| ***MTMR11*** | 1,12E-03 |
| ***MXRA5*** | 3,60E-04 |
| ***MYO1F*** | 1,80E-06 |
| ***MYOG*** | 5,32E-03 |
| ***MYRIP*** | 1,20E-03 |
| ***MZB1*** | 1,69E-02 |
| ***NACC2*** | 3,73E-01 |
| ***NAV1*** | 5,27E-03 |
| ***NCF4*** | 2,15E-03 |
| ***NCK2*** | 3,23E-05 |
| ***NCR3*** | 3,54E-05 |
| ***NFATC2*** | 5,75E-04 |
| ***NFE2L3*** | 4,08E-03 |
| ***NIPSNAP3B*** | 7,44E-04 |
| ***NKG7*** | 1,90E-06 |
| ***NLRC3*** | 1,13E-05 |
| ***NLRP3*** | 2,88E-03 |
| ***NOTCH2*** | 6,15E-03 |
| ***NPAS3*** | 5,46E-03 |
| ***NRADDP*** | 6,54E-04 |
| ***NRK*** | 3,40E-05 |
| ***NUP210*** | 1,49E-04 |
| ***OSBPL6*** | 4,50E-03 |
| ***OTUD7A*** | 3,88E-04 |
| ***P2RY2*** | 2,86E-04 |
| ***PAPLN*** | 1,92E-01 |
| ***PAQR5*** | 1,67E-02 |
| ***PARD6B*** | 2,17E-02 |
| ***PARP10*** | 2,19E-04 |
| ***PARP4*** | 9,26E-04 |
| ***PARVG*** | 6,95E-05 |
| ***PCDH20*** | 2,93E-04 |
| ***PCP4L1*** | 1,53E-02 |
| ***PCYOX1L*** | 6,09E-03 |
| ***PDE4D*** | 3,45E-04 |
| ***PDE4DIP*** | 3,78E-04 |
| ***PDE9A*** | 6,84E-03 |
| ***PDIA2*** | 3,43E-03 |
| ***PENK*** | 9,84E-05 |
| ***PHF21B*** | 2,12E-04 |
| ***PIP4K2A*** | 3,71E-05 |
| ***PITX1*** | 3,03E-03 |
| ***PKLR*** | 2,21E-03 |
| ***PLCB2*** | 3,60E-05 |
| ***PLD4*** | 1,89E-04 |
| ***PLXDC2*** | 3,22E-04 |
| ***POU2AF1*** | 1,96E-06 |
| ***PPM1M*** | 1,81E-03 |
| ***PPM1N*** | 5,60E-05 |
| ***PPP1CC*** | 6,39E-05 |
| ***PRAM1*** | 5,18E-06 |
| ***PRDX6*** | 3,48E-05 |
| ***PRF1*** | 5,20E-06 |
| ***PRLHR*** | 1,59E-04 |
| ***PRSS57*** | 6,55E-02 |
| ***PSD4*** | 6,04E-05 |
| ***PSTPIP1*** | 2,60E-07 |
| ***PSTPIP2*** | 4,08E-05 |
| ***PTDSS1*** | 2,93E-05 |
| ***PTK6*** | 1,20E-02 |
| ***PTPN22*** | 6,10E-07 |
| ***PTPN6*** | 2,42E-05 |
| ***PTPN7*** | 3,34E-07 |
| ***PTPRC*** | 2,12E-06 |
| ***PTPRCAP*** | 1,42E-07 |
| ***PTPRH*** | 3,14E-04 |
| ***PTPRO*** | 1,22E-05 |
| ***PTPRVP*** | 1,25E-04 |
| ***PVT1*** | 4,10E-03 |
| ***PYDC1*** | 1,11E-03 |
| ***PYGO1*** | 1,47E-02 |
| ***RAB30*** | 1,38E-03 |
| ***RAB37*** | 3,55E-04 |
| ***RAB8B*** | 6,65E-03 |
| ***RANBP17*** | 8,98E-03 |
| ***RAP1GAP2*** | 7,58E-05 |
| ***RARRES3*** | 4,69E-04 |
| ***RASAL3*** | 1,35E-06 |
| ***RASEF*** | 4,56E-04 |
| ***RASL10B*** | 2,20E-03 |
| ***RGS10*** | 4,23E-04 |
| ***RGS9BP*** | 3,80E-04 |
| ***RHEB*** | 2,12E-03 |
| ***RHOBTB1*** | 3,60E-03 |
| ***RHOD*** | 2,18E-04 |
| ***RHOH*** | 1,66E-07 |
| ***RLTPR*** | 2,67E-06 |
| ***RMI2*** | 4,31E-03 |
| ***RNF213*** | 2,43E-03 |
| ***RPH3A*** | 3,03E-03 |
| ***RPL27A*** | 1,91E-03 |
| ***RRAD*** | 3,00E-04 |
| ***RTN4RL1*** | 2,36E-05 |
| ***RUNX2*** | 1,38E-05 |
| ***RUNX3*** | 1,85E-06 |
| ***RYR1*** | 3,75E-04 |
| ***S1PR4*** | 1,10E-07 |
| ***SCG5*** | 1,28E-03 |
| ***SCML4*** | 2,29E-07 |
| ***SELPLG*** | 8,76E-06 |
| ***SEMA4D*** | 3,10E-06 |
| ***SFI1*** | 2,61E-04 |
| ***SFMBT2*** | 9,92E-04 |
| ***SH2D1A*** | 3,76E-07 |
| ***SH2D3A*** | 6,08E-07 |
| ***SH3TC1*** | 4,13E-04 |
| ***SHC4*** | 5,13E-03 |
| ***SIGLEC1*** | 3,61E-04 |
| ***SLA*** | 8,88E-06 |
| ***SLA2*** | 5,85E-04 |
| ***SLAMF1*** | 1,40E-03 |
| ***SLAMF8*** | 2,74E-06 |
| ***SLC25A34*** | 6,87E-03 |
| ***SLC4A8*** | 4,77E-05 |
| ***SLC7A7*** | 7,74E-05 |
| ***SLFN12L*** | 2,01E-04 |
| ***SLIT2*** | 2,48E-06 |
| ***SLITRK4*** | 1,61E-04 |
| ***SMOC2*** | 6,34E-03 |
| ***SNX20*** | 1,34E-06 |
| ***SOCS1*** | 6,70E-04 |
| ***SORCS1*** | 2,02E-02 |
| ***SP140*** | 4,18E-05 |
| ***SP140L*** | 3,12E-06 |
| ***SPATA24*** | 9,26E-02 |
| ***SPC24*** | 1,22E-03 |
| ***SRGAP2*** | 1,24E-03 |
| ***SRRM3*** | 9,25E-03 |
| ***STAG3*** | 1,46E-03 |
| ***STAT1*** | 1,81E-02 |
| ***SUSD4*** | 4,23E-03 |
| ***SYNGAP1*** | 2,06E-03 |
| ***SYPL2*** | 4,88E-03 |
| ***SYT7*** | 2,75E-04 |
| ***SYTL1*** | 1,93E-06 |
| ***TACSTD2*** | 9,81E-05 |
| ***TBC1D10C*** | 5,73E-08 |
| ***TCEANC*** | 9,46E-03 |
| ***TESC*** | 8,70E-03 |
| ***TFCP2L1*** | 1,32E-03 |
| ***TGFBR2*** | 1,53E-02 |
| ***THY1*** | 1,22E-03 |
| ***TIFAB*** | 2,72E-06 |
| ***TLCD1*** | 7,64E-05 |
| ***TLR6*** | 2,92E-03 |
| ***TMC8*** | 2,11E-08 |
| ***TMEM132E*** | 3,45E-04 |
| ***TMEM171*** | 6,78E-03 |
| ***TMEM189*** | 9,18E-03 |
| ***TMPRSS3*** | 1,14E-04 |
| ***TNFAIP8L2*** | 8,22E-04 |
| ***TNFRSF19*** | 1,80E-03 |
| ***TNFSF13B*** | 5,97E-05 |
| ***TNFSF14*** | 7,38E-05 |
| ***TNIP3*** | 2,13E-05 |
| ***TNNI2*** | 3,13E-05 |
| ***TP53INP1*** | 2,80E-04 |
| ***TPD52*** | 3,38E-03 |
| ***TRAF1*** | 1,25E-03 |
| ***TRAF3IP3*** | 1,24E-07 |
| ***TRAF5*** | 3,13E-04 |
| ***TRERF1*** | 2,14E-05 |
| ***TRIM14*** | 2,42E-05 |
| ***TRIM4*** | 6,60E-05 |
| ***TRIM66*** | 1,02E-02 |
| ***TRIP13*** | 4,13E-01 |
| ***TRPC4*** | 1,73E-03 |
| ***TRPM2*** | 1,24E-04 |
| ***TSPAN5*** | 1,11E-03 |
| ***TTC24*** | 2,17E-04 |
| ***TTYH1*** | 4,52E-04 |
| ***TUSC1*** | 2,11E-04 |
| ***TYROBP*** | 5,35E-04 |
| ***UBASH3A*** | 2,74E-06 |
| ***UBD*** | 1,66E-04 |
| ***UMOD*** | 3,19E-03 |
| ***VAC14*** | 1,89E-05 |
| ***WDFY4*** | 1,39E-05 |
| ***WIPF1*** | 5,76E-04 |
| ***WNT3A*** | 1,66E-03 |
| ***WSCD1*** | 7,61E-03 |
| ***WWC1*** | 1,66E-02 |
| ***XAF1*** | 4,63E-04 |
| ***XRCC3*** | 2,67E-03 |
| ***ZACN*** | 2,13E-03 |
| ***ZFHX4*** | 8,48E-03 |
| ***ZMYND15*** | 1,99E-04 |
| ***ZNF214*** | 7,81E-03 |
| ***ZNF217*** | 5,49E-05 |
| ***ZNF831*** | 2,09E-07 |
| ***ZNRD1-AS1*** | 4,86E-04 |
