# Supplementary material for: Whole-Genome Cardiac DNA Methylation Fingerprint and Gene Expression Analysis Provide New Insights in the Pathogenesis of Chronic Chagas Disease Cardiomyopathy
Source: Clin Infect Dis. 2017 May 30;65(7):1103–11. doi: 10.1093/cid/cix506 (PMC5849099; doi:10.1093/cid/cix506)
Supplement: Supplementary_table_7_20170516 [file cix506_suppl_supplementary_table_7_20170516.docx]

**Supplementary table 7:** Results of the pyrosequencing done on human heart tissue samples.

| **Gene** | **Target** | **Chr** | **Position** | **Illumina chip p value** | **Pyromark assay** | **Pyromark**  **p value** | **STATUT** |
| --- | --- | --- | --- | --- | --- | --- | --- |
| ***RUNX3*** | **cg01103597** | **1** | **25242147** | 5,21E-08 | PM00640892 | 5,59E-02 | not confirmed |
|  | **cg04221877** | **1** | **25257515** | 6,47E-09 | PM00628453 | 9,87E-13 | confirmed |
|  | **cg18087266** | **1** | **25257629** | 8,05E-08 | PM00641004 | 5,92E-09 | confirmed |
|  | **cg24463471** | **1** | **25257978** | 4,32E-08 | PM00641088 | 1,49E-09 | confirmed |
|  | **cg24006721** | **1** | **25258225** | 2,96E-09 | PM00641095 | 5,14E-09 | confirmed |
|  | **cg00929376** | **1** | **25258236** | 2,80E-08 | PM00641095 | 1,54E-08 | confirmed |
|  | **cg03171924** | **1** | **25258332** | 6,77E-08 | PM00641179 | 2,60E-03 | confirmed |
|  | **cg20670361** | **1** | **25258679** | 2,23E-08 | PM00628509 | 3,75E-05 | confirmed |
|  | **cg19774846** | **1** | **25292225** | 8,44E-08 | PM00641431 | 3,00E-08 | confirmed |
|  |  |  |  |  |  |  |  |
| ***HLA-DPA1*** | **cg20907136** | **6** | **33041221** | 5,31E-08 | PM00643146 | 3,53E-05 | confirmed |
|  | **cg09321817** | **6** | **33041343** | 8,70E-08 | PM00643118 | 3,85E-08 | confirmed |
|  | **cg22483030** | **6** | **33043849** | 8,68E-08 | PM00643202 | 1,05E-04 | confirmed |
|  | **cg13349035** | **6** | **33048310** | 4,92E-09 | PM00631218 | 2,14E-07 | confirmed |
|  |  |  |  |  |  |  |  |
| ***PTPRCAP*** | **cg03559915** | **11** | **67201998** | 7,44E-09 | PM00630623 | 4,30E-10 | confirmed |
|  | **cg02685484** | **11** | **67203360** | 2,53E-08 | PM00643888 | 2,54E-09 | confirmed |
|  | **cg05353099** | **11** | **67203544** | 2,91E-09 | PM00630581 | 1,77E-08 | confirmed |
|  | **cg02423817** | **11** | **67203661** | 1,09E-11 | PM00630791 | 6,56E-05 | confirmed |
|  | **cg20792833** | **11** | **67205195** | 7,94E-09 | PM00630868 | 2,75E-07 | confirmed |
|  | **cg23468927** | **11** | **67206263** | 1,08E-08 | PM00630819 | 4,72E-09 | confirmed |
|  | **cg12044599** | **11** | **67206308** | 4,67E-10 | PM00630763 | 2,32E-09 | confirmed |
|  | **cg02740606** | **11** | **67206418** | 5,81E-08 | PM00630707 | 6,11E-10 | confirmed |
|  | **cg17690322** | **11** | **67206434** | 5,25E-09 | PM00630707 | 1,60E-09 | confirmed |
|  |  |  |  |  |  |  |  |
| ***LTB*** | **cg02402436** | **6** | **31540051** | 3,12E-08 | PM00642901 | 7,50E-08 | confirmed |
|  | **cg17709873** | **6** | **31540456** | 4,02E-08 | PM00642957 | 8,42E-09 | confirmed |
|  | **cg26348243** | **6** | **31540461** | 2,87E-09 | PM00631561 | 2,55E-06 | confirmed |
|  | **cg01360627** | **6** | **31544931** | 6,20E-08 | PM00643069 | 1,90E-10 | confirmed |
|  | **cg20477259** | **6** | **31544960** | 6,28E-08 | PM00643069 | 1,45E-04 | confirmed |
|  | **cg04472685** | **6** | **31545473** | 5,11E-08 | PM00642999 | 6,25E-08 | confirmed |
|  | **cg16167809** | **6** | **31556255** | 7,11E-08 | PM00631589 | 5,99E-05 | confirmed |
|  | **cg16280132** | **6** | **31540459** | 3,82E-11 | PM00631561 | 2,83E-07 | confirmed |
|  |  |  |  |  |  |  |  |
| ***UBD*** | **cg02149189** | **6** | **29521138** | 1,44E-08 | PM00630952 | 2,02E-07 | confirmed |
|  | **cg22617773** | **6** | **29521751** | 1,15E-08 | PM00642600 | 4,61E-01 | not confirmed |
|  | **cg14278853** | **6** | **29521756** | 2,60E-08 | PM00642600 | 8,80E-01 | not confirmed |
|  |  |  |  |  |  |  |  |
| ***PTPN6*** | **cg23147227** | **12** | **7060187** | 3,74E-09 | PM00631463 | 2,44E-10 | confirmed |
|  | **cg21249754** | **12** | **7060206** | 1,64E-08 | PM00631463 | 9,26E-09 | confirmed |
|  | **cg14311559** | **12** | **7060257** | 1,57E-09 | PM00631379 | 3,38E-09 | confirmed |
|  | **cg26842815** | **12** | **7060263** | 1,04E-09 | PM00631379 | 8,27E-10 | confirmed |
|  | **cg19693177** | **12** | **7060569** | 1,29E-09 | PM00631414 | 2,51E-11 | confirmed |
|  | **cg04234016** | **12** | **7062109** | 3,91E-08 | PM00643916 | 7,69E-03 | not confirmed |
|  | **cg15264991** | **12** | **7066563** | 4,23E-08 | PM00643986 | 7,51E-04 | not confirmed |
|  | **cg24437859** | **12** | **7066614** | 3,93E-08 | PM00644042 | 4,72E-09 | confirmed |
|  |  |  |  |  |  |  |  |
| ***KCNA4*** | **cg10387551** | **11** | **30038615** | 9,23E-09 | PM00632079 | 8,22E-06 | confirmed |
|  | **cg17714025** | **11** | **30038619** | 5,11E-11 | PM00632093 | 1,66E-05 | confirmed |
|  | **cg15044957** | **11** | **30038672** | 1,98E-09 | PM00631995 | 1,32E-02 | not confirmed |
|  | **cg08490115** | **11** | **30038675** | 1,54E-09 | PM00631995 | 8,71E-04 | not confirmed |
|  | **cg15310492** | **11** | **30038677** | 7,25E-11 | PM00631995 | 6,22E-04 | not confirmed |
|  | **cg05756220** | **11** | **30038685** | 1,09E-10 | PM00632135 | 4,94E-03 | not confirmed |
|  | **cg22685409** | **11** | **30038692** | 2,55E-11 | PM00631995 | 2,96E-01 | not confirmed |
|  |  |  |  |  |  |  |  |
| ***LSP1*** | **cg04085571** | **11** | **1872753** | 5,68E-08 | PM00631225 | 8,04E-04 | confirmed |
|  | **cg04666911** | **11** | **1872756** | 8,29E-08 | PM00631225 | 2,43E-05 | confirmed |
|  | **cg19280572** | **11** | **1873884** | 1,48E-08 | PM00631029 | 9,08E-04 | confirmed |
|  | **cg07237979** | **11** | **1874017** | 2,45E-08 | PM00643447 | 1,23E-07 | confirmed |
|  | **cg05305434** | **11** | **1874049** | 2,83E-08 | PM00643447 | 4,51E-10 | confirmed |
|  | **cg09060789** | **11** | **1884988** | 8,58E-09 | PM00631141 | 5,27E-07 | confirmed |
|  |  |  |  |  |  |  |  |
| ***PENK*** | **cg04127342** | **8** | **57358130** | 4,11E-09 | PM00628782 | 1,21E-06 | confirmed |
|  | **cg19414741** | **8** | **57358240** | 2,39E-09 | PM00628495 | 4,83E-06 | confirmed |
|  | **cg12877723** | **8** | **57358312** | 1,49E-08 | PM00628537 | 8,66E-01 | not confirmed |
|  | **cg04598121** | **8** | **57358505** | 8,36E-10 | PM00628439 | 8,62E-03 | not confirmed |
|  | **cg10397440** | **8** | **57359258** | 8,29E-08 | PM00645197 | 4,75E-05 | confirmed |
|  | **cg16072688** | **8** | **57360711** | 8,64E-08 | PM00645239 | 8,69E-06 | confirmed |
|  |  |  |  |  |  |  |  |
| ***ZNF217*** | **cg07617814** | **20** | **52198164** | 2,67E-08 | PM00629216 | 4,19E-03 | not confirmed |
|  | **cg12032027** | **20** | **52198225** | 6,36E-08 | PM00644889 | 2,47E-07 | confirmed |
|  | **cg01692482** | **20** | **52198378** | 8,15E-10 | PM00629244 | 1,52E-07 | confirmed |
|  | **cg09029902** | **20** | **52199594** | 3,34E-08 | PM00644847 | 2,39E-10 | confirmed |
|  | **cg22164891** | **20** | **52199729** | 1,55E-08 | PM00629391 | 4,08E-08 | confirmed |
|  | **cg02535674** | **20** | **52226352** | 1,39E-08 | PM00629265 | 2,91E-03 |  |
|  |  |  |  |  |  |  |  |
| ***CD6*** | **cg13014558** | **11** | **60738971** | 3,46E-08 | PM00643727 | 2,14E-09 | confirmed |
|  | **cg09902130** | **11** | **60739178** | 1,23E-08 | PM00643804 | 9,91E-10 | confirmed |
|  | **cg09153080** | **11** | **60739183** | 3,19E-09 | PM00643804 | 2,61E-09 | confirmed |
|  |  |  |  |  |  |  |  |
| ***RANBP17*** | **cg23873669** | **5** | **170289421** | 1,40E-08 | PM00629461 | 4,66E-09 | confirmed |
|  | **cg07848601** | **5** | **170289430** | 9,45E-08 | PM00629461 | 1,09E-09 | confirmed |
|  | **cg26844246** | **5** | **170736277** | 7,50E-10 | PM00629503 | 8,18E-07 | confirmed |
|  |  |  |  |  |  |  |  |
| ***TRAF3IP3*** | **cg20694619** | **1** | **209929496** | 3,00E-09 | PM00642096 | 5,18E-09 | confirmed |
|  | **cg05959508** | **1** | **209929512** | 5,20E-09 | PM00642096 | 5,09E-07 | confirmed |
|  | **cg01997629** | **1** | **209929622** | 8,76E-08 | PM00642229 | 1,11E-09 | confirmed |
|  | **cg09802688** | **1** | **209942066** | 5,81E-08 | PM00642285 | 2,17E-09 | confirmed |
|  |  |  |  |  |  |  |  |
| ***PTPN7*** | **cg25754958** | **1** | **202130692** | 1,78E-08 | PM00628908 | 7,77E-11 | confirmed |
|  | **cg15027815** | **1** | **202131184** | 5,70E-09 | PM00629062 | 4,25E-08 | confirmed |
|  |  |  |  |  |  |  |  |
| ***PRF1*** | **cg09914304** | **10** | **72362292** | 2,50E-09 | PM00629734 | 1,42E-07 | confirmed |
|  | **cg23364656** | **10** | **72362715** | 3,52E-08 | PM00643643 | 2,69E-06 | confirmed |
|  | **cg02374486** | **10** | **72362809** | 1,23E-08 | PM00629832 | 2,62E-06 | confirmed |
|  | **cg15293582** | **10** | **72362866** | 1,60E-09 | PM00630238 | 2,34E-06 | confirmed |
|  |  |  |  |  |  |  |  |
| ***SMOC2*** | **cg10176110** | **6** | **168841653** | 5,45E-14 | PM00640178 | 1,81E-08 | confirmed |
|  | **cg01568319** | **6** | **168841907** | 4,52E-09 | PM00640199 | 5,68E-03 | not confirmed |
|  | **cg02540477** | **6** | **168842035** | 3,79E-08 | PM00640206 | 1,08E-05 | confirmed |
|  | **cg05076914** | **6** | **168842302** | 5,79E-10 | PM00643286 | 1,03E-07 | confirmed |
|  | **cg11612345** | **6** | **168842491** | 6,03E-08 | PM00643342 | 2,60E-05 | confirmed |
|  |  |  |  |  |  |  |  |
| ***KCNIP4*** | **cg07695835** | **4** | **20985984** | 6,42E-09 | PM00640073 | 1,53E-06 | confirmed |
|  | **cg06482428** | **4** | **21950173** | 3,17E-10 | PM00640003 | 5,96E-03 | not confirmed |
|  |  |  |  |  |  |  |  |
| ***SLC7A7*** | **cg25570278** | **14** | **23306509** | 4,09E-09 | PM00644189 | 1,65E-07 | confirmed |
|  | **cg16119835** | **14** | **23306850** | 1,56E-09 | PM00644217 | 6,68E-03 | not confirmed |
|  |  |  |  |  |  |  |  |
| ***FCHO1*** | **cg02639359** | **19** | **17862017** | 9,68E-08 | PM00644707 | 1,51E-08 | confirmed |
|  | **cg08358907** | **19** | **17877508** | 4,79E-10 | PM00644749 | 1,59E-09 | confirmed |
|  | **cg06336535** | **19** | **17877528** | 1,38E-08 | PM00644777 | 6,44E-07 | confirmed |
|  |  |  |  |  |  |  |  |
| ***ATP2A3*** | **cg07005444** | **17** | **3820796** | 1,33E-09 | PM00641368 | 1,33E-07 | confirmed |
|  | **cg10095954** | **17** | **3848324** | 2,72E-09 | PM00641354 | 4,42E-09 | confirmed |
|  | **cg25738176** | **17** | **3848506** | 1,59E-08 | PM00641347 | 6,45E-07 | confirmed |
|  |  |  |  |  |  |  |  |
| ***CD247*** | **cg21161394** | **1** | **167408709** | 2,22E-11 | PM00641487 | 2,14E-05 | confirmed |
|  | **cg12446199** | **1** | **167408841** | 3,12E-08 | PM00641914 | 3,42E-05 | confirmed |
|  | **cg07786657** | **1** | **167487633** | 1,50E-08 | PM00641200 | 1,83E-08 | confirmed |
|  |  |  |  |  |  |  |  |
| ***LST1*** | **cg01360627** | **6** | **31544931** | 6,20E-08 | PM00643069 | 1,90E-10 | confirmed |
|  | **cg20477259** | **6** | **31544960** | 6,28E-08 | PM00643069 | 1,45E-04 | confirmed |
|  | **cg04472685** | **6** | **31545473** | 5,11E-08 | PM00642999 | 6,25E-08 | confirmed |
|  | **cg16167809** | **6** | **31556255** | 7,11E-08 | PM00631589 | 5,99E-05 | confirmed |

**STATUT :** When a significant p value was obtained either on methylation array or by pyrosequencing we are indicating that the association of the CpG site to the disease is confirmed.
