# Supplementary material for: Whole-Genome Cardiac DNA Methylation Fingerprint and Gene Expression Analysis Provide New Insights in the Pathogenesis of Chronic Chagas Disease Cardiomyopathy
Source: Clin Infect Dis. 2017 May 30;65(7):1103–11. doi: 10.1093/cid/cix506 (PMC5849099; doi:10.1093/cid/cix506)
Supplement: Supplementary_table_8_20170516 [file cix506_suppl_supplementary_table_8_20170516.docx]

**Supplementary table 8:** Statistical analyses of the results of the gene reporter assays done on HEK293 and AC16 cell lines.

| **Genes** | **Statistical comparisons** | **Expression level variations** | **P values** |
| --- | --- | --- | --- |
| **HEK293 cell line** | | | |
| *KCNA4* | vector /w insert versus vector w/ insert | 243% | 2,29E-15 |
|  | vector w/ insert versus vector w/ methylated insert | 41% | 8,54E-11 |
|  | vector /w insert versus vector w/ methylated insert | 103% | 9,24E-10 |
|  |  |  |  |
| *KCNIP4* | vector /w insert versus vector w/ insert | 43% | 1,03E-04 |
|  | vector w/ insert versus vector w/ methylated insert | 76% | 2,20E-11 |
|  | vector /w insert versus vector w/ methylated insert | 66% | 2,86E-08 |
|  |  |  |  |
| *LSP1* | vector /w insert versus vector w/ insert | 100% | 3,70E-10 |
|  | vector w/ insert versus vector w/ methylated insert | 89% | 9,71E-13 |
|  | vector /w insert versus vector w/ methylated insert | 78% | 6,79E-10 |
|  |  |  |  |
| *RUNX3* | vector /w insert versus vector w/ insert | 389% | 1,25E-12 |
|  | vector w/ insert versus vector w/ methylated insert | 55% | 9,93E-11 |
|  | vector /w insert versus vector w/ methylated insert |  | 6,17E-14 |
|  |  |  |  |
| *PENK* | vector /w insert versus vector w/ insert | 12% | 2,21E-01 |
|  | vector w/ insert versus vector w/ methylated insert | 171% | 9,09E-11 |
|  | vector /w insert versus vector w/ methylated insert | 138% | 9,17E-10 |
|  |  |  |  |
| *SMOC2* | vector /w insert versus vector w/ insert | 1232% | 4,14E-12 |
|  | vector w/ insert versus vector w/ methylated insert | 69% | 3,26E-09 |
|  | vector /w insert versus vector w/ methylated insert | 2144% | 1,32E-13 |
|  |  |  |  |
| *CD6* | vector /w insert versus vector w/ insert | 25% | 6,86E-03 |
|  | vector w/ insert versus vector w/ methylated insert | 53% | 1,10E-06 |
|  | vector /w insert versus vector w/ methylated insert | 65% | 7,31E-08 |
|  |  |  |  |
| *HLA-DPA1* | vector /w insert versus vector w/ insert | 984% | 1,08E-10 |
|  | vector w/ insert versus vector w/ methylated insert | 20% | 1,33E-02 |
|  | vector /w insert versus vector w/ methylated insert | 772% | 3,29E-14 |
|  |  |  |  |
| *PRF1* | vector /w insert versus vector w/ insert | 541% | 1,16E-07 |
|  | vector w/ insert versus vector w/ methylated insert | 92% | 4,99E-07 |
|  | vector /w insert versus vector w/ methylated insert | 1133% | 2,68E-12 |
|  |  |  |  |
| *PTPN7* | vector /w insert versus vector w/ insert | 13% | 6,27E-01 |
|  | vector w/ insert versus vector w/ methylated insert | 581% | 1,51E-08 |
|  | vector /w insert versus vector w/ methylated insert | 489% | 1,37E-08 |
|  |  |  |  |
| *PTPRCAP* | vector /w insert versus vector w/ insert | 171% | 5,48E-05 |
|  | vector w/ insert versus vector w/ methylated insert | 25% | 8,25E-02 |
|  | vector /w insert versus vector w/ methylated insert | 105% | 1,34E-02 |
|  |  |  |  |
| *TRAF3IP3* | vector /w insert versus vector w/ insert | 80% | 2,15E-01 |
|  | vector w/ insert versus vector w/ methylated insert | 52% | 1,38E-01 |
|  | vector /w insert versus vector w/ methylated insert | 13% | 8,32E-01 |
|  |  |  |  |
| **AC16 cell line** | | | |
| *KCNA4* | vector /w insert versus vector w/ insert | 151% | 1,17E-12 |
|  | vector w/ insert versus vector w/ methylated insert | 22% | 1,99E-03 |
|  | vector /w insert versus vector w/ methylated insert | 207% | 4,03E-12 |
|  |  |  |  |
| *KCNIP4* | vector /w insert versus vector w/ insert | 590% | 1,68E-13 |
|  | vector w/ insert versus vector w/ methylated insert | 65% | 1,71E-10 |
|  | vector /w insert versus vector w/ methylated insert | 140% | 5,71E-09 |
|  |  |  |  |
| *LSP1* | vector /w insert versus vector w/ insert | 35% | 5,28E-05 |
|  | vector w/ insert versus vector w/ methylated insert | 17% | 1,05E-02 |
|  | vector /w insert versus vector w/ methylated insert | 46% | 1,58E-06 |
|  |  |  |  |
| *RUNX3* | vector /w insert versus vector w/ insert | 4111% | 8,30E-16 |
|  | vector w/ insert versus vector w/ methylated insert | 14% | 3,27E-03 |
|  | vector /w insert versus vector w/ methylated insert | 4683% | 2,35E-15 |
|  |  |  |  |
| *PENK* | vector /w insert versus vector w/ insert | 17% | 2,97E-01 |
|  | vector w/ insert versus vector w/ methylated insert | 36% | 6,77E-02 |
|  | vector /w insert versus vector w/ methylated insert | 47% | 3,88E-05 |
|  |  |  |  |
| *SMOC2* | vector /w insert versus vector w/ insert | 103% | 2,82E-08 |
|  | vector w/ insert versus vector w/ methylated insert | 137% | 1,48E-13 |
|  | vector /w insert versus vector w/ methylated insert | 381% | 1,37E-13 |
|  |  |  |  |
| *CD6* | vector /w insert versus vector w/ insert | 29% | 1,07E-04 |
|  | vector w/ insert versus vector w/ methylated insert | 34% | 1,38E-05 |
|  | vector /w insert versus vector w/ methylated insert | 72% | 3,89E-09 |
|  |  |  |  |
| *HLA-DPA1* | vector /w insert versus vector w/ insert | 660% | 4,24E-12 |
|  | vector w/ insert versus vector w/ methylated insert | 64% | 4,55E-11 |
|  | vector /w insert versus vector w/ methylated insert | 176% | 1,90E-09 |
|  |  |  |  |
| *PRF1* | vector /w insert versus vector w/ insert | 58% | 1,65E-07 |
|  | vector w/ insert versus vector w/ methylated insert | 11% | 8,64E-02 |
|  | vector /w insert versus vector w/ methylated insert | 75% | 7,84E-10 |
|  |  |  |  |
| *PTPN7* | vector /w insert versus vector w/ insert | 92% | 4,85E-06 |
|  | vector w/ insert versus vector w/ methylated insert | 2754% | 2,17E-01 |
|  | vector /w insert versus vector w/ methylated insert | 120% | 4,77E-01 |
|  |  |  |  |
| *PTPRCAP* | vector /w insert versus vector w/ insert | 3214% | 1,15E-12 |
|  | vector w/ insert versus vector w/ methylated insert | 15% | 1,03E-02 |
|  | vector /w insert versus vector w/ methylated insert | 2705% | 9,70E-14 |
|  |  |  |  |
| *TRAF3IP3* | vector /w insert versus vector w/ insert | 139% | 5,25E-09 |
|  | vector w/ insert versus vector w/ methylated insert | 26% | 1,30E-03 |
|  | vector /w insert versus vector w/ methylated insert | 201% | 5,84E-12 |

Two kinds of cells were used for transfections (HEK293 cells and AC16 cells). Human embryonic kidney cells (HEK293) are a specific cell line originally derived from human embryonic kidney, widely used in transfection experiments. The AC16 Human cardiomyocyte cell line is a proliferating cell line that was derived from the fusion of primary cells from adult human ventricular heart tissues with SV40 transformed human fibroblasts. This last cell line is more adapted to study cardiac gene expression and function, during normal development and in pathological condition. For luciferase activity, for each construct, the experiments were done in quadruplicates and the experiments were repeated four times.
